# Chemical Engineering Laboratory Projects in Student Teams in Real Life and Transformed Online: Viscose Fiber Spinning and Characterization

**DOI:** 10.1021/acs.jchemed.8b00790

**Published:** 2021-03-29

**Authors:** Michael Weißl, Gregor Kraft, Josef Innerlohinger, Tiina Nypelö, Stefan Spirk

**Affiliations:** † Institute of Bioproducts and Paper Technology, Graz University of Technology, Inffeldgasse 23, 8010 Graz, Austria; ‡ Lenzing AG, Werkstrasse 2, 4860 Lenzing, Austria; § Department of Chemistry and Chemical Engineering and Wallenberg Wood Science Center, Chalmers University of Technology, SE-412 96 Gothenburg, Sweden

**Keywords:** Upper-Division Undergraduate, Graduate Education/Research, Chemical Engineering, Organic Chemistry, Hands-On Learning/Manipulatives, Laboratory Instruction

## Abstract

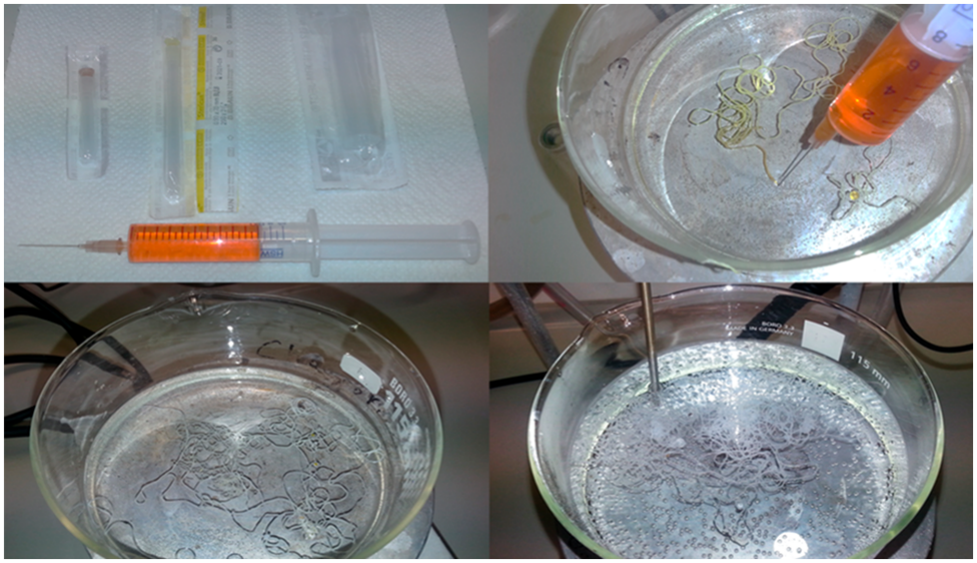

Chemical engineering education comprises a complexity of technical skills that include learning processes that are currently relevant in industry. Despite being a rather old industrial process, the manufacturing of viscose fibers still accounts for the major fraction of all human-made cellulosic fibers worldwide. Here we describe a laboratory setup to introduce chemistry and engineering students into the principles of cellulose fiber spinning according to the viscose process. The setup for fiber spinning is kept simplistic and allows the experiments to be performed without professional spinning equipment. However, all of the steps are performed analogously to the industrial process. The professional setting in process and chemical engineering involves work on projects and in teams. Hence, we have incorporated the fiber spinning laboratory experiment in the context of working in teams on projects. We will also present our experience on transferring a real-life laboratory experiment online, as this is required at times that online education is preferred over real-life teaching.

## Introduction and Relevance of the Technical Topic

Project-based and inductive learning are typically implemented to increase the engagement of students in learning via tasks that mimic real-world problems with a non-school-like agenda and technological challenges that are relevant in industry.^1−3^ Here, an industrially important and large-volume fiber spinning process is incorporated into a project in chemistry and engineering education aiming to provide project topics that have a bearing on current industrial settings. In contrast to previous reports on demonstrating cuprammonium-based rayons in a high school laboratory project^4^ where the experiments were proposed to be conducted by an instructor,^5^ here we present a procedure that can be fully or partly executed by students.

The history of human-made wood-based fibers dates back to 1884, when Svan dissolved nitrocellulose and then injected this solution into a regeneration bath to produce cellulose fibers with a wonderful shine, the first artificial silk. In the years to come, a variety of other processes were introduced and commercialized, such as the cuprammonium rayon process and—with large success—the viscose silk process.^6^ Soon after the discovery of viscose silk by Cross, Bewan, and Beadle in 1892, it conquered the markets and replaced artificial silks made from nitrocellulose and cuprammonium.^7,8^ In detail, Cross, Bewan, and Beadle explored the reaction of alkali cellulose and carbon disulfide to give cellulose xanthate. They realized that the produced cellulose xanthate is very soluble in dilute sodium hydroxide solution and can be converted after a processing step to cellulose fibers and films by exposure to an acidic bath. By the 1930s, a wide range of production facilities in the U.S. had started producing either viscose or other rayons.^9^ In general, the term rayon refers to a wide range of human-made cellulose fibers from different processes.^10^ Among those, the viscose process is nowadays the most important process to manufacture human-made wood-based fibers, with production volumes of several million tons per year.^11,12^ Although the basic principle is still the same as it was at the end of the 19th century, much progress has been made in the preparation of the alkali cellulose, the removal of undesired impurities, and the procedures to obtain spinning dopes suitable for the production of high-quality fibers.^13−15^ Besides progress in fiber/film manufacturing, also the processes to recycle the used chemicals and remove them from the air have been improved significantly.^16,17^ For instance, emissions by the largest viscose manufacturer in Europe, Lenzing AG, is at an extremely low level, which is a prerequisite considering that the plant site is located close to residential areas.^18^ Since the demand for fibers is steadily growing and cotton plantations are more or less at the limit of potential land use, the only way to close the gap is to focus on wood-based fibers.^19^ The viscose process is certainly a part of the solution to this challenge.

## Pedagogical Goals

### Nontechnical Skills

Mastery of technical skills is not the sole requirement for professionals who are being educated in engineering programs in universities. Additionally, it is required that the students are able to function as a part of a professional setting. Such work at the current time is carried out by teams in which individual team members are carefully selected on the basis of their competences but also on their abilities to work in teams. Working in teams relies on functioning team dynamics. Team work in projects has been described to consist of the phases of forming, storming, norming, performing, and adjourning.^20,21^ In the forming phase, the team may experience consensus, but pushing the boundaries of the consensus later on can lead to a storming phase. The storming phase is critical for the team to reach a status where individual competencies and ways of working are established and the team can reach an optimized performing phase. Adjourning is a part of time-limited projects that is characteristic of the work in student teams in education.

Working in teams during degree studies is vital for the development of soft skills such as communication, problem solving, and time management.^22^ Hence, the fiber spinning experiment is planned to be conducted in small groups (four or five students) with interaction between the students. Indeed, mastery of soft skills can be decisive in future recruitments of the students after their studies. Sharma^23^ confirmed that in recruitment for technical jobs, communication skills were rated as the most important by 72%, followed closely by teamwork at 66% and then time management at 60%.

Diversity in teams has been identified to be favorable, as diversity in competencies and personalities can create a synergy in teams.^24^ Within this context, the technical background of the students was considered in the teams’ composition: chemistry, biorefinery engineering, and material science students were distributed to enable diverse teams. The aim of executing the project in teams was that it would enable more efficient learning via sharing of knowledge and tasks than working individually. We also aimed to provide the students a setting emulating real life and hypothesized that it would lead to motivation.

The intended learning outcomes relevant to the nontechnical skills are the following:

- To be able to work in small teams (four to five students) in a project and to exchange experiences between teams
- To acquire knowledge on how to effectively perform literature research, store and manage the literature, and extract the required information in a synopsis.

### Technical Skills

**Scheme 1:**
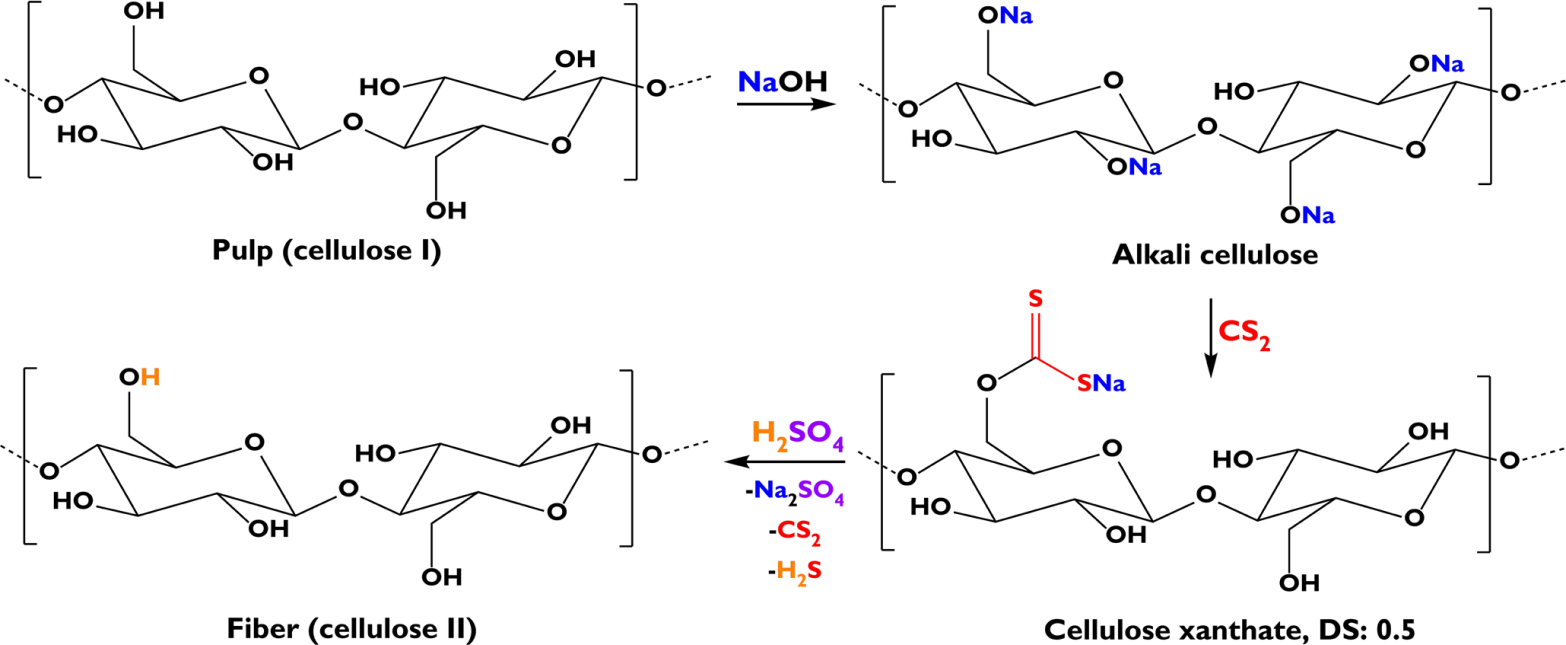
Simplified Description of the Main Steps in the Viscose Process: Synthesis of Alkali Cellulose from a Cellulose Source, Xanthation, and the Final Regeneration Back to Cellulose Using Sulfuric Acid (DS is the Degree of Substitution).

The laboratory project has the intention to demonstrate the basic steps of viscose fiber manufacturing (Scheme 1). The first step is the conversion of a cellulose source (e.g., pulp or cotton) to alkali cellulose. The obtained fibrous material is then reacted with CS_2_ to form an alkaline-soluble compound, so-called cellulose xanthate (CX). The last step in viscose fiber spinning is the conversion of CX back to cellulose, a process commonly called “regeneration”.

These reactions are the basis for the achieving the pedagogical goals and are complemented by theoretical background (see the Supporting Information) for each individual step. The intended learning outcomes relevant to the technical skills are the following:

- Understanding challenges in cellulose processing and how to overcome them
- Acquiring practical knowledge in wood-based fiber manufacturing
- Exploring the morphological properties of the spun wood-based fibers
- Distinguishing between the supramolecular structures of viscose fibers and natural ones (e.g., cotton)
- Documenting the observations made during the different steps of fiber spinning

In this laboratory project, the assignment and project question were given by the supervisor. For the course in real life, the assignment was to spin viscose fibers, analyze the product, and report the outcome. In the virtual project, the project assignment and questions were to design this particular laboratory course on viscose fiber spinning from scratch.

### Teacher Perspective: Transferring Real-Life Laboratory Exercises Online

The shift toward online education requires rethinking of procedures in our teaching routines. While lectures can be implemented with a similar quality online, the design and execution of virtual laboratory courses poses challenges. Work in the laboratory is an essential part of training for a chemist, and a virtual experience can hardly compensate for a real-life experience. Here, we adapted the fiber spinning project into a virtual laboratory course. It has been executed during one semester so far, and it is a work in progress for iteration. Since direct transformation of the laboratory activities online is unlikely to provide the same learning as the real-life training, we executed a different concept. The challenge in the real-life version of the laboratory course for the students is to master the different steps of the viscose process in an experimental manner. This requires knowledge of chemistry, process engineering, and laboratory safety as well as nontechnical skills such as problem solving and the ability to interact efficiently with team members. In the virtual version, rather than doing experiments, the students needed to design this particular laboratory course on viscose fiber spinning from scratch. This involved literature search, safety aspects, and how to design the experiments so that they can be performed in a laboratory at a university. The challenge for the teacher is to motivate the students who are working remotely. Regular and highly structured digital meetings with the students are essential, as the feeling of being overwhelmed at the beginning might demotivate them. We have seen that in this phase it is important to connect the students so that they can interchange their experiences in their quest to master the challenge and have the feeling that they are working as a team toward the final goal, even if they are operating remotely. It is essential to provide constructive and timely feedback to the students, as literature research on viscose fiber spinning requires the students to look for old literature that is not easily available online. Here, the balance between guiding the students while letting them drive the project is something that needs to be addressed in the future. In a later stage of the course, the knowledge about the technical aspects of the project needs to be provided by the teacher to the students. In order to achieve maximum learning impact, the spinning setup used for the real-life laboratory course was shown to the students individually after the virtual course had been completed.

### The Cognitive Process

In the virtual version, the assignment given to the students does not include details of the technical execution, and the students need to design a fiber spinning process using a literature screening approach. The virtual version is an open-ended project and leads to a unique product. The real-life version has some level of predefined outcome, yet the project-based learning experience stems from the real-life mimicking of team work in projects.

In order to understand the cognitive process in the real-life projects, we need to consider the backgrounds of the students. In the course that the exercise was implemented, they were from three different programs, namely, chemistry, biorefinery engineering, and advanced materials science. The tasks in the fiber spinning project are given in detail on alkali cellulose synthesis, xanthation, fiber spinning, and characterization. Therefore, the project comprises unit operations dominated by chemistry, process, materials, and characterization. While, for example, for a chemistry student the reactions are easy to grasp, the concepts of a process are more challenging. Processing and unit operation thinking is trained in the biorefinery curriculum, giving this competence to the students with the biorefinery background. However, the detailed chemical reactions are a challenge for that class of students. The advanced materials science students are trained in multidisciplinary aspects with a focus on material structure–property relationships, making the analysis approachable to them within the viscose fiber spinning laboratory project. However, in order to be able to perform the entire exercise in the laboratory, the students need to use the diversity in the knowledge base of their team.

## Evaluation

### Nontechnical Skills

The nontechnical skills did not contribute directly to the grade. The communication ability to summarize findings, interpret their meaning, and provide alternative strategies and problem solution capability during the laboratory work and the laboratory report was included in the assessment of the technical skills (see the next section).

### Technical Skills

The grading of the laboratory project consisted of 100 points and comprised a starting exam (30%), performance in the laboratory (30%), and the final report (40%). These evaluated events take place in a sequence, and the students receive feedback after each section is finished. The completed section is a gate to the next one.

The starting exam (written or oral, depending on the number of students) takes place prior to the beginning of the experimental work in the laboratory. The exam covers the theoretical background and the experimental procedures, including safety aspects and hazards of the involved chemicals. The performance of the students in the laboratory (30%) is assessed by the supervisor and is based on understanding the process steps and order, chemical reactions in each step, and ability to follow safety guidelines. The final report was evaluated for structure (a template was given), accuracy of description of the process and reactions, clarity of presentation, and match with the actual events in the laboratory.

The emphasis in the evaluation of all steps is to examine whether the students can follow and understand the process chain starting from pulp followed by alkali cellulose, cellulose xanthate, and regeneration to cellulose and how this relates to the working principle of fiber spinning. Lack of knowledge in details, e.g., concerning side or secondary reactions in xanthation, is not punished in the course if the overall concept of fiber spinning including the main steps is well understood.

### Virtual Version

The grading of the virtual laboratory course consisted of the following tasks in a sequence: (1) preparation of a literature database (10%) that was evaluated by quantity and quality; (2) description of the three most significant scientific publications on viscose spinning (20%) in 800–1000 words, for which the assessment criteria were relevance to the lab course design and ability to summarize concisely the main results; and (3) design of the laboratory experiment (70%), which was evaluated on the basis of quality and involved a detailed description of the required chemicals, glassware, devices, procedures, and processes, the feasibility of performing the experiments in a standard chemistry laboratory, and a consideration of safety aspects. In an ideal report, the main chemical reactions as well as potential hazards were identified, and adequate protective gear was proposed. The description of the process comprised also the post-treatment of the fibers as well as their analysis.

## Experimental Overview

### Laboratory Experiment

The experimental parts necessary for the production of viscose fibers in a laboratory course are in principle the same steps that are necessary in large-scale viscose fiber production. However, simplified equipment can be used, and it is not necessary to perform all of the steps in the course, providing flexibility while adjusting to the skills and background of students. The options range from a half-day laboratory project, where the focus is only on the main part (the fiber spinning), to a laboratory project that lasts 4 days, where all of the important steps of wood-based fiber production are practiced. In the following, an overview of the individual units (four laboratory periods, each for 5 hours) of the laboratory course on viscose fiber spinning will be given; detailed information on every step is available in the Supporting Information. The optimum group size is two or three students for each experiment.

#### Alkali Cellulose Reaction

The reaction of cellulose to give alkali cellulose is performed. A native cellulose source (pulp, cotton, or paper) is stirred in an 18 wt % sodium hydroxide solution for 2 h. After this, the excess sodium hydroxide solution is removed from the cellulose by pressing. Afterward, the cellulose is aged overnight.^25,26^

#### Xanthation

The aged alkali cellulose is stirred, and carbon disulfide is added dropwise. After addition of the carbon disulfide, the xanthation takes place, and the white alkali cellulose fibers are converted to an orange, sticky pulp. The cellulose xanthate can then be dissolved in a 4 wt % sodium hydroxide solution under constant stirring and cooling, resulting in the so-called viscose solution. After the cellulose xanthate is completely dissolved, the viscose solution is subjected to aging before it can be used for fiber spinning.^25^

#### Fiber Spinning

The aged cellulose xanthate is injected into a 10 wt % sulfuric acid solution at 50 °C. For the injection, different experimental setups are used. The basic spinneret consists of a simple syringe with an injection needle, while a more sophisticated one employs a syringe pump with different spinning nozzles. The fibers are collected, for example, with a simple stirring bar when injected by hand or by a rotating polypropylene cylinder operated by a laboratory stirrer in the case a syringe pump is used.

#### Characterization

In the last part of the course, the changes in chemistry during the viscose process are observed by Infrared (IR) spectroscopy,^27^ and the effects of different injection needles, injection speeds, and ways of injection (manual injection by hand or controlled pressure by a syringe pump) on the fiber quality are studied.

### Virtual Laboratory Exercise

The interaction of the virtual laboratory course was organized using digital meeting software (Webex). Meetings took place at regular intervals, at least once per week over a period of 8 weeks. In the first session, the students were introduced to the topic, and the challenge was presented to them. This included (1) literature research on the viscose process, with a focus on experimental design and analysis; (2) creation of a literature database using Citavi or Mendeley; (3) summary of the three most relevant papers in 800–1000 words, using their own language, including justification of relevance to the design of the laboratory course; (4) design of the laboratory course using the collected literature; and (5) delivery of a laboratory course procedure containing the steps of the viscose process, including safety measures, that could be used by university students at the Master’s level. During the weekly digital meetings, every student had the opportunity to express problems and obstacles impeding the progress in solving the final challenge. These issues were discussed in the group from the very beginning, allowing for the generation of a team experience. Further guiding support was provided by the teacher.

## Hazards

Depending on the individual substeps of the laboratory experiment, attention has to be paid to potentially occurring risks. General protective measures include the use of protective glasses, gloves, and a laboratory coat. The safety data sheets for the used chemicals have to be studied beforehand. In the alkali cellulose synthesis, the 18 wt % sodium hydroxide solution has to be handled with care because of its high alkalinity. The xanthation reaction uses small amounts of carbon disulfide, a highly flammable and potentially harmful compound. The use of a fume hood is obligatory, and any type of operation should be performed using syringes to avoid any exposure. For the regeneration of the cellulose xanthate, the spinning bath should also be placed in a fume hood because during regeneration traces of hydrogen sulfide gas are formed. H_2_S is toxic and potentially harmful to environment and health. Additionally, the work with the 10 wt % sulfuric acid requires attention. After the fibers have been washed, they can be handled and investigated without any safety equipment.

## Results and Discussion

### Nontechnical Skills

University students are familiar with working on projects involving technical challenges with a clearly defined aim. They master intrinsically the basic concepts of communication in writing and time management to allow the tasks to be finalized. Most often the challenges arise from working in teams and especially management of interpersonal relationships. This can define the input that an individual is motivated to give to the exercise. How to work in teams and the communication between the students in this laboratory project were enabled but not supported with specific activities. The supervisor monitors the teamwork and facilitates it. However, we foresee that especially performing the project as a virtual laboratory course creates a need for first observing how the students are able to work together virtually and later on by developing a systematic support for teamwork that will be a subject of iterations as we learn more about team dynamics in virtual settings.

### Technical Skills in the Real-Life Laboratory Course

In our standard laboratory protocol, the students focus on optical and microscopy observations regarding fiber shape and structure when needles with different diameters are used for the injection or when the injection speed of the syringe pump is changed. The fiber diameter has to be determined from the optical microscopy images, and the changes in diameter have to be explained. The second important part in the analytical section is to acquire IR spectra of all of the cellulose derivatives appearing in the process and to explain the changes in structure and the ongoing chemical reactions using the absorption bands in the infrared spectra.

However, if available, many other characterization techniques like tensile testing for the fibers or viscometric studies of the ripening of the cellulose xanthate solution can be used.

#### Determination of the Cellulose Content

The determination of the cellulose content in the spinning dope is an easy way to characterize the dope without any further analytical instrument needed, as described in the Supporting Information. The absolute cellulose content could then be compared with the theoretical cellulose content based on the amount of cellulose source used. Normally the absolute and theoretical cellulose contents fit well: in the provided example, 3.5% was calculated and 3.6% was experimentally determined.^28^

#### Microscopy Images and Fiber Diameter

**Figure 1:**
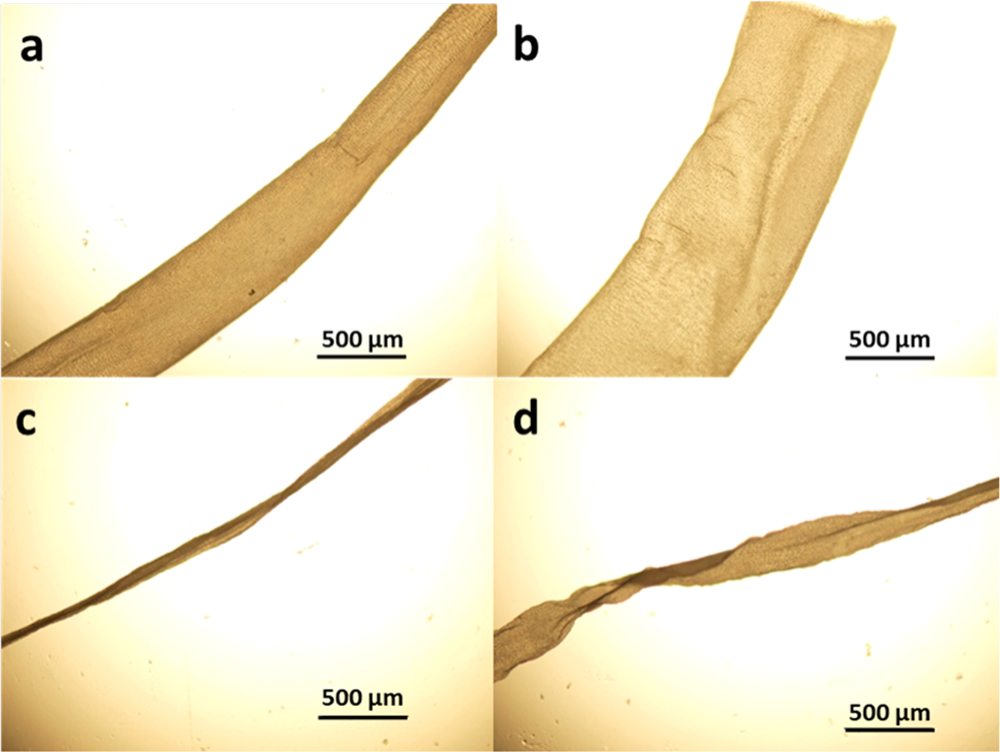
Fibers obtained either through manual injection of the viscose (a, b) or from continuous injection via the syringe pump (c, d). For the fiber spinning, different injection needles with diameters of 450 μm (a, c) and 800 μm (b, d) were used.

The fibers presented in Figure 1 exhibited large differences in terms of fiber diameter, fiber morphology, and uniformity. The spinning dope used was the same in all of the experiments, and only a few variations such as the injection mode used (manual or continuous), the collection of the fibers (rotating fiber collector), and the diameter of the employed injection needles (450 or 800 μm) were explored. The manually injected fibers showed a relatively large variation in the fiber diameter and uniformity. The diameters were around 450 and 820 μm, respectively, which were in the same range as the diameters of the injection needles used since no additional force was applied for the collection of the fibers. The large variation in the diameters was caused by fluctuations of the caused injection pressure. In contrast, the fibers formed by continuous injection and collected under tension appeared quite uniform, and the variation in the diameters was much smaller. Diameters of approx. 60 μm for the thin needle and approx. 250 μm for the thicker needle were manufactured.

#### ATR-IR Spectra

**Figure 2:**
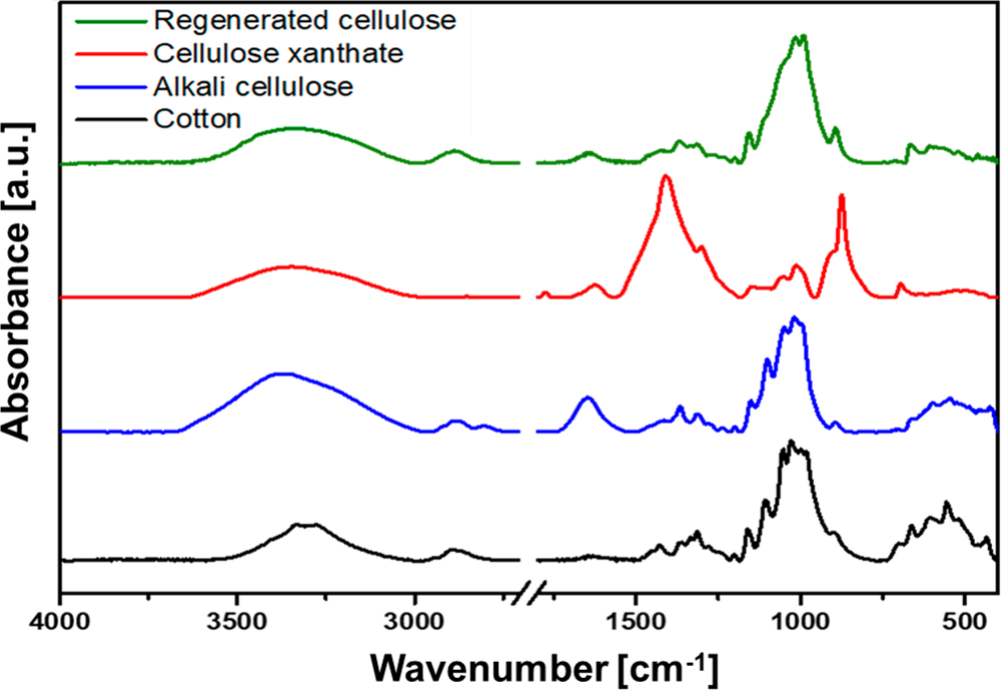
ATR-IR spectra of the different modifications and derivatives of cellulose that appear during the steps of the viscose process, starting with cotton (cellulose I) and proceeding via alkali cellulose and cellulose xanthate to regenerated cellulose (cellulose II).

IR spectroscopy is a widely available and commonly used method to study the reactions of cellulose and its derivatives. The used starting material, highly purified cotton fibers, exhibit an IR spectrum characteristic of cellulose I (Figure 2). It features broad bands from 3600 to 3100 cm^–1^ (ν_OH_) and from 3000 to 2850 cm^–1^ (C–H stretching vibrations), a series of small weak bands in the region of 1430 to 1150 cm^–1^ (C–O–H bending at 1430 cm^–1^, C–H deformation at 1372 cm^–1^, OH in-plane deformation at 1330 and 1200 cm^–1^), strong and overlapping bands from 1160 to 950 cm^–1^ (asymmetric C–O–C vibration at 1155 cm^–1^, symmetric C–O vibration at 1060 cm^–1^, and C–O stretching at 1035 cm^–1^), and a small band at 899 cm^–1^ (C–O–C valence vibration).^29^

The alkali cellulose spectrum differs significantly from the cotton spectrum. The broad band at 3600–3000 cm^–1^ is shifted from 3500 to 3650 cm^–1^ in the alkali cellulose spectrum, and the form changes to a single band. The increased intensity at 1640 cm^–1^ indicates the presence of additional OH stretching and deformation vibrations caused by remaining water stored in the alkali cellulose.

In the case of CX, the interpretation of the IR spectrum is not that straightforward. Significant amounts of primary and secondary reaction products can be identified. These products can be identified as CS_2_ (ν_C=S_ at 1520 cm^–1^), sodium sulfide (1420 and 920 cm^–1^), and sodium trithiocarbonate (1670, 1427, 925, and 885 cm^–1^).^11,30^ The remaining bands at 1452 and 1382 cm^–1^ as well as a weak band at 2725 cm^–1^ can be assigned to NaOH, which is present from the dissolution of CX. Since NaOH is highly hygroscopic, the water peak at 1640 cm^–1^ is pronounced as well. The C–S and C=S vibrations for CX have been reported in the region around 900 cm^–1^ and between 1050 and 1250 cm^–1^ and interfere with these products as well as with vibrations of the pyranose ring, which also shows absorption bands in this range.^31^ Therefore, an unambiguous assignment is not possible.

After regeneration and extensive washing of the spun fibers, again a cellulose spectrum is obtained, which is denoted as cellulose II.

### Virtual Laboratory Project

For the students, it was a unique experience to design a laboratory course. Usually, the students follow a more-or-less strict procedure in laboratory courses that has been designed by others. This procedure contains many meta-aspects (e.g., available infrastructure, glassware, safety measures) of which most students were not aware. Furthermore, the involvement in designing teaching content engages students.^32,33^ The use of literature database programs created a new experience that is useful for later courses in the study programs of the students. The assignment to sum up complex papers in short paraphrases and justify their relevance to the laboratory course improved their writing skills. The work required intense interaction in a virtual manner with the other students and with the teacher.

Although the course was virtual, the workload was high and was underestimated by the students in the beginning. As the relevant literature for the viscose process is from the 1920–1970s, some of the content is not easily available on the online and requires the use of databases such as SciFinder and ISIS Web of Knowledge. Some of the relevant literature was not available at all in electronic form and required a visit to the university library. The feedback from the students was positive. However, they mentioned that in the beginning they were overwhelmed, as this was a new approach for them. In addition, the virtual format slows down the social interaction with others, meaning that the teacher needs to provide extra support and motivation for the students to team up. The supervisor enabled this by setting up regular team meetings online where the students and teams could interconnect and share their experiences as well as their approach toward solving problems, particularly on literature search. The supervisor needs to seek a moderating role in such meetings and needs to actively motivate students to talk to each other to enable student–student interactions. As the laboratory course progressed, students got more self-confident on the challenge, and in the end they considered it a valuable experience and enrichment to their education program.

## Conclusions

Working in teams in a project is a necessary skill for chemists and engineers and hence should be part of university education. Here, teamwork was executed on a project that had a technical challenge relevant to industry. The procedure for the synthesis of cellulose xanthate and the following spinning of regenerated cellulose fibers is an easy-to-follow laboratory experiment that does not require expensive materials or extraordinary characterization techniques. An additional benefit of this experiment is that it is not necessary to have extensive and specialized knowledge in the production of human-made fibers through the viscose process. The time frame for the course is flexible, depending on which steps are performed with the students and which materials are prepared beforehand. A basic knowledge of organic chemistry and a laboratory equipped with the standard tools, safety precautions, and a motivated teacher are required. The learning outcomes for the students involve practical knowledge on fiber spinning using the viscose process, including all of the required steps and analytical procedures. They are aware of the difficulties in shaping and processing of cellulose, including the challenges to make fibers of good quality. The success of the laboratory project was reflected by comparison of the results of the exams before the practical work started and the laboratory reports.

The virtual laboratory course represents an alternative to get students familiarized with the viscose process. The virtual version of the laboratory course differs from the real-life experience as it targets the design of a laboratory course from scratch, requiring the students to think outside their normal laboratory course bubble, where the materials are readily available and a supervisor is in control of the course of experiments. The exercise is definitely more challenging not only for the university students but also the university teacher, as it requires constant efforts and focus on motivation and student interaction in a virtual setting. Although the virtual course cannot fully replace hands-on training, it is a viable way to provide the students with useful knowledge in times where laboratory access is restricted.

The students were able to fulfill the intended learning outcomes directed toward the soft skills that constitute the ability to work in teams and acquire, process, and communicate information. The performance was not assessed in the final grade, but learning of these outcomes is deemed a necessity to arrive at the submission of a report that can be accepted from the technical side. Achieving the technical learning outcome shows variation within students that was reflected in the grade of the laboratory exercise. That is typical in the case of mastering a complex process in a technical university teaching setup.

## Supporting Information Available

Laboratory handouts for the individual steps in the fiber spinning process (including aims, theoretical background, schemes, and photographs, Figures S1–S5); instructor notes; examples of exam questions, including a key; ATR-IR spectra of cellulose I (starting material), alkali cellulose, cellulose xanthate, and regenerated spun cellulose fibers (Figures S6–S9) and assignment of the most important bands; procedure for the virtual laboratory course, including material that was provided to the students (PDF, DOCX)
