# Supplementary material for: Chemical Engineering Laboratory Projects in Student Teams in Real Life and Transformed Online: Viscose Fiber Spinning and Characterization
Source: J Chem Educ. 2021 Mar 29;98(5):1776–82. doi: 10.1021/acs.jchemed.8b00790 (PMC8161680; doi:10.1021/acs.jchemed.8b00790)
Supplement: Supplementary file 2 — ed8b00790_si_002.docx [file ed8b00790_si_002.docx]

**Supporting Information**

**Chemical engineering project laboratory work in teams in real life and transformed online: viscose fiber spinning and characterization**

Michael Weißl^†^, Gregor Kraft^‡^, Josef Innerlohinger^‡^, Tiina Nypelö^¥^ and Stefan Spirk^†^*

†Graz University of Technology, Institute of Bioproducts and Paper Technology, Inffeldgasse 23, 8010 Graz; Austria.

‡Lenzing AG, Werkstrasse 2, 4860 Lenzing, Austria.

^¥^Chalmers University of Technology, Department of Chemistry and Chemical Engineering, Gothenburg, Sweden and Wallenberg Wood Science Center, Chalmers University of Technology, Gothenburg, Sweden.

Correspondence:

Prof. Dr. Stefan Spirk

[stefan.spirk@tugraz.at](mailto:stefan.spirk@tugraz.at)

+43 (316) 873 – 30763

**Table of Contents**

1. Real Life Laboratory course S3

1.1. Instructor notes S3

1.2. Laboratory Hand Outs S5

1.2.1. Part 1: Alkali cellulose synthesis S5

1.2.2. Part 2: Xanthation S9

1.2.3. Part 3: Fiber spinning S16

1.2.4. Part 4: Characterization S23

1.3. Examples for exam questions S26

1.4. Key for exam questions S28

2. Virtual laboratory course S35

2.1. Instructor notes S35

2.2. Laboratory Hand Outs S36

2.2.1. Part 1: Alkali cellulose synthesis S36

2.2.2. Part 2: Xanthation S38

2.2.3. Part 3: Fiber spinning S42

2.3. Tasks for the virtual laboratory course S44

References S46

**1. Real Life Laboratory course**

**1.1. Instructor notes**

We performed the fiber spinning experiments in different master courses in our chemistry, biorefinery engineering and advanced materials science curricula. The group size was 4 to 5 students, depending on the course type and its aims. Cellulose xanthate was only prepared in the advanced courses and that course lasted for a period of one week with 5 hours daily laboratory work, including preparation of alkali cellulose, xanthation and fiber spinning. In the basic courses of fiber spinning, students did not synthesize the cellulose xanthate but used ready-made samples for fiber spinning. Here, the duration of the course was two days with 5 hours of daily laboratory work including characterization. In the light version of the laboratory course (2-3 hours), just fiber spinning is demonstrated and the characterization part is reduced to analyzing the IR spectra and judging microscopy images which both had been acquired beforehand.

Before start of the laboratory work, an examination was performed in order to see whether students understood the tasks of the work and whether the students were aware of the safety precautions required for the different experiments. It is important to introduce the students into potential hazards of the involved lab experiments This particularly comprises the reaction of the alkali cellulose with the CS_2_ which must be performed in a fume hood. We recommend this part of the course only to be performed in advanced laboratory courses where students have a profound knowledge in chemical engineering or chemistry. Further, the spinning of the fibers into the sulfuric acid must also be performed in a fumehood as traces of H_2_S and CS_2_ may be formed during the regeneration step. We recommend to use just small amounts of the samples as indicated in the experimental procedures below. For the practical work, crucial steps involve the spinning with a syringe into a sulfuric acid bath. This step needs to be shown by the teacher in advance, with a focus on the proper attachment of the needle to the syringe during the spinning process to avoid detachment during spinning.

We started this laboratory exercises in 2015 and in the meanwhile, ca 200 students did these experiments in the various courses. Feedback from the students has been very positive. The experience to create fibers is something students will memorize for a long time.

**1.2. Laboratory Hand Outs**

**1.2.1. Part 1: Alkali cellulose synthesis**

**Aims of the task**

Understand processes during conversion of cellulose to alkali cellulose and to connect these to fiber spinning requirements

**Theoretical background^1^**

The formation of alkali cellulose is the first step in the manufacturing of viscose fibers. The main reactant is sodium hydroxide to give the sodium salt of cellulose. In industry, standard dissolving pulp is mixed with 18-20 wt.% NaOH to form a ca. 5 wt.% suspension. This suspension is exposed to a temperature between 25 and 55 °C for some hours. At this stage, the low molecular weight fraction of cellulose as well as the hemicelluloses dissolve and are removed by pressing the alkali cellulose. The removal of hemicellulose is essential for the resulting fiber quality since hemicellulose xanthate (which would be produced in the next step if they were not removed) are much more unstable than cellulose xanthate. This subsequently leads to formation of gels, thereby deteriorating the filterability of the obtained solutions. Further, hemicellulose incorporation into the cellulose fiber matrix during spinning leads to fiber with lower tenacity. After pressing, the alkali cellulose typically contains less than 3.5 wt.%. hemicelluloses, for speciality products even contents of below 1.6 wt.% are required. The pressing step also removes excess NaOH, which reacts with the CS_2_ in the xanthation step. After pressing, the alkali cellulose contains 7-8 wt.% bound and ca 9 % adsorbed NaOH. Then the alkali cellulose is subjected to pre-ripening, i.e. storage at 30-55°C for a period between 10 and 30 hours under defined humidity control. During the pre-ripening a wealth of reactions take place, hydrogen bonds of the crystalline parts of the pulp are broken up and depolymerization of the cellulose macromolecules occurs. The most important reactions are depicted in Scheme S1.


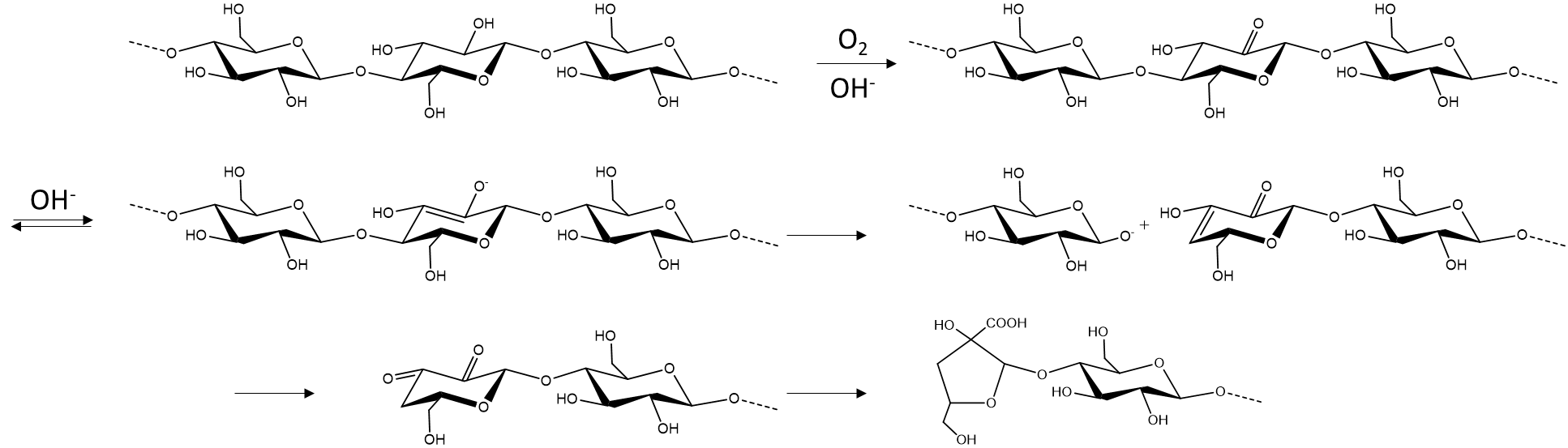


**Scheme S1**. Reactions during the processing of pulp to alkali cellulose

For fiber spinning, it is required to have a rather homogenous degree of polymerization (DP) for the material of interest. The average DP decrease from the range 850-600 to 500-350.

**Materials needed:**

Cellulose source (cotton, pulp) 7 g No hazards

Sodium hydroxide 27 g Harmful (strong base)

DI water 123 ml No hazards

Laboratory stirrer 1

1000 ml flask 1

Protective gear (glasses, gloves, lab coat) for everybody

**Before start:**

Make sure that everything you need is organized and available.

Be sure, that the lab stirrer and the flask are safely installed.

Inform yourself how to deal with strong bases and what to do in case of an accident.

**To consider for the laboratory report:**

Save a gram of your cellulose source for the IR spectroscopy

Note the changes in the cellulose appearance during the reaction

Describe the differences in optical appearance between cellulose, wet alkali cellulose (after pressing) and dried alkali cellulose

**Task:**

Take 7 g of the cellulose (pulp is preferable, cotton is also possible), cut it into small pieces, and transfer it into the 500 ml flask. Dissolve 27 g sodium hydroxide in 123 ml DI water to prepare an 18 wt.% NaOH solution. After the NaOH has been completely dissolved, add the solution to your flask. Mount the lab stirrer and the flask, before you start adding the pulp into the NaOH solution. After two hours of stirring, transfer the alkali cellulose on a kitchen paper and remove the excess sodium hydroxide solution by pressing the alkali cellulose with your hands. The pressed alkali cellulose is pulled apart into small pieces and dried overnight.
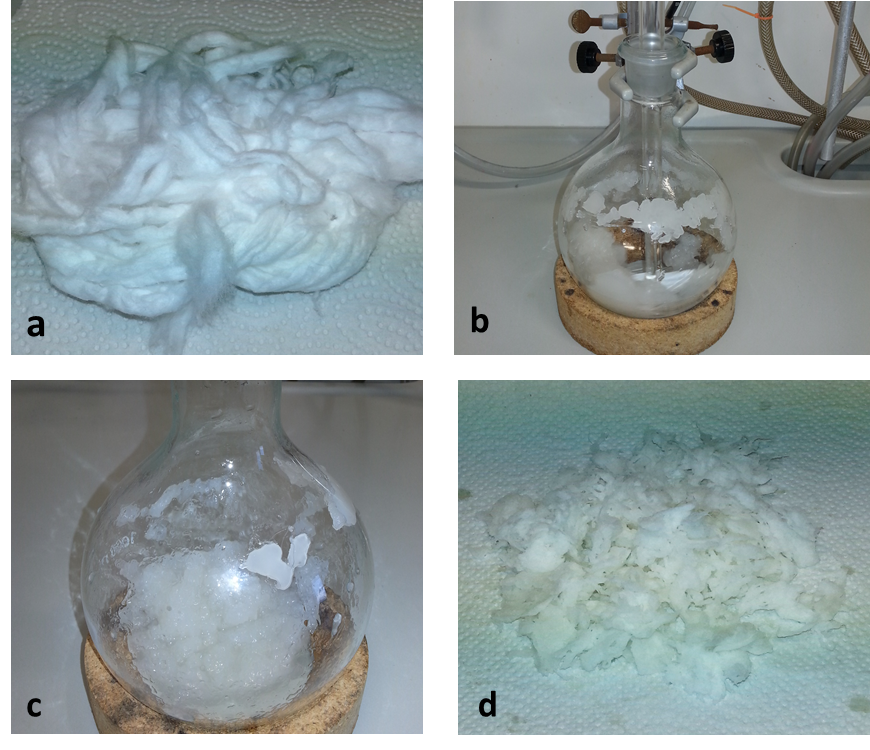
Figure S1 depicts the process of the alkali cellulose synthesis as described above.

**Figure S1.** Illustration of alkali cellulose preparation. a: starting material-a cellulose source, b: stirring of the cotton fibers in 18% NaOH solution, c: alkali cellulose before pressing, d: alkali cellulose after pressing.

**1.2.2. Part 2: Xanthation**

**Aims of the task**

Connect the processes happening during xanthation of alkali cellulose to fiber spinning parameters.

Understand importance of spinning dope preparation

**Theoretical background^2-7^**

The next step in viscose synthesis is xanthation. In this step, the alkali cellulose is converted to the sodium cellulose xanthate. In industry, this exothermic reaction is done in huge cylinders where the CS_2_ is reacted in vaporous state (either injected as gas or vaporized in vessel; reaction time ca 100 minutes, temperature needs to be kept between 25-30°C by cooling). The degree of xanthation is expressed in industrial terms as the so called gamma number. The gamma number is related to the degree of substitution (DS) via equation (1):

$\gamma=100*DS\left( X \right)$ (1)

In industry, cellulose xanthate with a gamma value of around 50 is usually manufactured for fiber spinning, which means that in average just 1 out of two AGU is substituted with xanthates. Higher gamma numbers can be realized if subsequent additions of CS_2_ are performed, in lab scale even full substitution (gamma 300) can be realized.

In fact, a wealth of reactions proceed at this step, which can be classified into primary, secondary and side reactions. Therefore 30 up to 50 wt.% of CS_2_ related to cellulose are needed in industrial scale to accomplish the reaction to the desired product. Primary reactions refer to those reactions where the desired cellulose xanthate is formed (eq 2-5).

CS_2_ + Cell-O^-^Na^+^ → Cell-OCS_2_^-^Na^+^  (2)

CS_2_ + Na^+^OH^-^ → HS-CSO^-^Na^+^ (3)

HCS_2_O^-^Na^+^ + Cell-OH → Cell-OCS_2_^-^Na^+^ + H_2_O (4)

HCS_2_O^-^Na^+^ + Na^+^OH^-^ → CS_2_O^2-^ + 2 Na^+^ (5)

The secondary reactions are those that occur with the formed products from the primary reactions such as the thiol and the dithiocarbonate. The latter for instance reacts with CS_2_ to form COS, which in turn reacts with NaOH to the carbonate under release of thiols, which then

are further converted to the trithiocarbonate (eq 6-10).

CS_2_ + CS_2_O^2-^ → COS + CS_3_^2-^ (6)

COS + 3 OH^-^ → CO_3_^2-^ + SH^-^ + H_2_O (7)

CS_2_ + SH^-^ → CS_3_H^-^ (8)

CS_3_H^-^ + OH^-^ → CS_3_^2-^ + H_2_O (9)

CS_2_O^2-^ + 2 OH^-^ → 2 SH^-^ + CO_3_^2^ (10)^-^

In addition, the reaction of NaOH and CS_2_ (side reactions) leads to a variety of different sulfur containing compounds such as sodium carbonate, sodium thiolate, sodium trithiocarbonate, hydrogen sulfide, and sodium sulfide. (eq 11-14)

CS_2_ + 4 NaOH → Na_2_CO_3_ + 2 NaHS + H_2_O (11)

2 CS_2_ + 4 NaOH → Na_2_CO_3_ + Na_2_CS_3_ + H_2_S + H_2_O (12)

3 CS_2_ + 6 NaOH → 2 Na_2_CS_3_ + Na_2_CO_3_ + 3 H_2_O (13)

5 CS_2_ + 12 NaOH → Na_2_S + 2 Na2CO3 + 3 Na_2_CS_3_ + 6 H_2_O (14)

From these reactions, it is also obvious why the pressing step after alkali cellulose preparation is so important. Every mol of NaOH can consume CS_2_ which is an economically important factor. After the xanthation has been finished, the cellulose xanthate is diluted with NaOH, a usual ratio is 1:4 (1 part alkali cellulose, 4 parts NaOH) to form the so called viscose. Then the viscose (before xanthate) is transferred to a tank, where the ripening takes place for a period of 18-30 hours at a temperature smaller than 18°C. The conditions for the ripening are crucial: cellulose xanthate decomposes at higher temperatures but also at the lower temperatures transxanthation reactions occur, which are, however, beneficial for fiber spinning. In order to obtain mechanically strong fibers, the macromolecules must be of similar size and in addition, they must be able to align with each other under flow conditions. This can be realized much easier when the xanthate substitution pattern along the cellulose chain is homogeneous. Here, it is important to know that the thermodynamically most stable position for the xanthate is the C6 position; therefore any rearrangements, i.e. transxanthation reactions (either inter- or intramolecular) lead to preferred substitution at C6. In addition, also a partial cleavage of the xanthate groups occurs whereas hydrolysis is ca. times faster at position C2 and C3 compared to C6, thereby also contributing to a final cellulose xanthate spinning dope where the xanthate groups are preferentially located at C6. An overview is presented in Scheme S5.


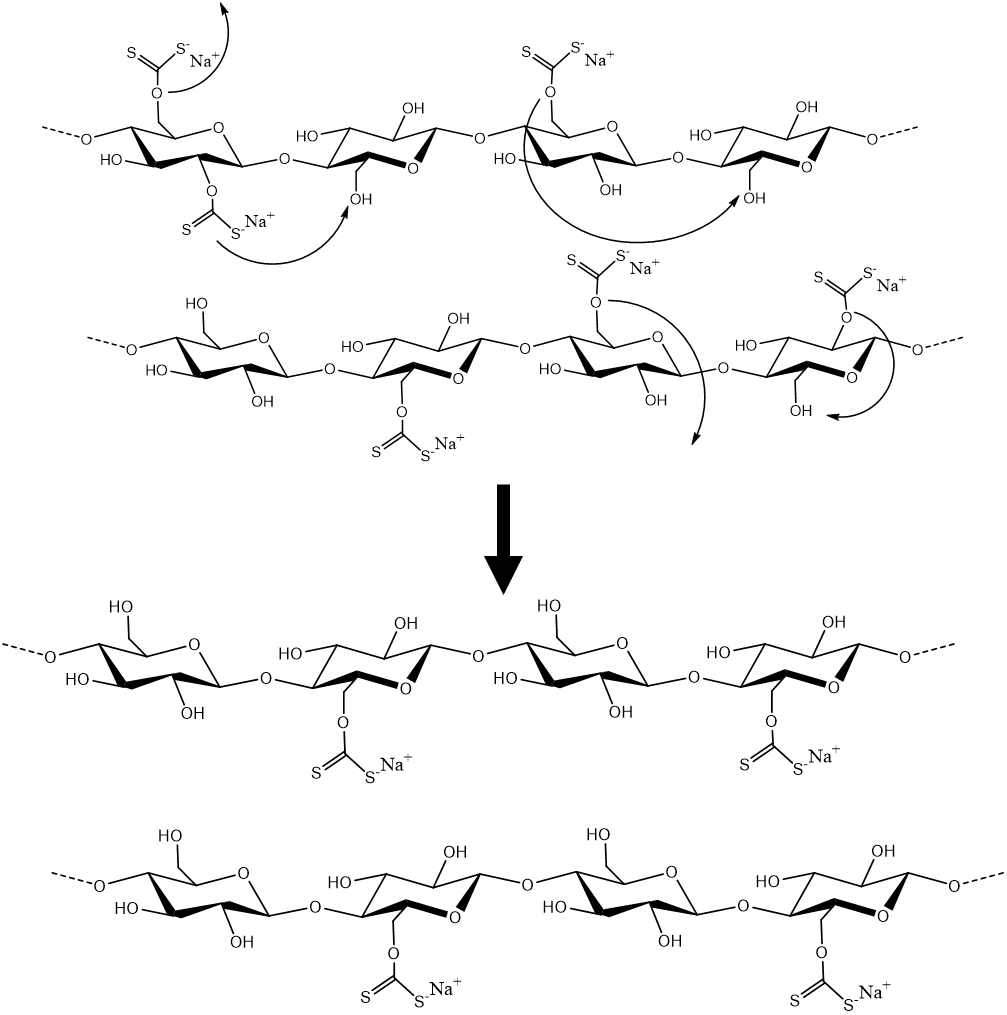


**Scheme S2**. Overview of cleavage and transxanthation reactions

**Materials needed:**

Alkali cellulose 4.5 g (dry mass cellulose) Low hazards

Sodium hydroxide 4 g Harmful (strong base)

DI water 100 ml No hazards

Carbon disulfide 7.1 ml (9 g) Harmful (toxic)

Water bath 1

250 ml 3 neck flask 1

Dropping funnel or septum 1

Laboratory stirrer 1

Reflux cooler 1

Heating plate 1

Protective gear (glasses, gloves, lab coat) for everybody

**Before start:**

Make sure that everything you need is organized and available.

Ensure yourself that your apparatus is mounted the right way.

Inform yourself about the correct handling and the dangers of CS_2_ by reading the SDS accurately.

**To consider for the laboratory report:**

Save a bit of the remaining alkali cellulose for the IR spectroscopy

Observe and explain the ongoing reactions during xanthation.

**Task:**

Determine the weight of the overnight dried alkali cellulose to calculate the dry mass cellulose content of your alkali cellulose (15):

$\frac{weight of cellulose}{weight of alkalicellulose} x 100=cellulose content \left[ \% \right]$ (15)

Transfer the amount of alkali cellulose, which equals 4.5 g pure cellulose into a 250 ml three-necked flask, add the stirrer and fix everything. Deposit a water bath below the flask and pre heat the alkali cellulose to 30 °C. Install a reflux condenser on one bottom of the flask and a dropping funnel on another one. (Figure S2a) After pre-heating the alkali cellulose, transfer CS_2_ (2 g per gram of cellulose) into the drooping funnel using a syringe and start adding the CS_2_ dropwise to the alkali cellulose under constant stirring. It should be mentioned here that for the lab scale synthesis a larger excess than in industrial production was used to accomplish for faster reaction. After addition is completed, continue stirring for three more hours. The starting material is finally converted into an orange sticky pulp (Figure S2b). This material is then dissolved in 100 ml of a 4% NaOH solution as shown in Figure S2c (25 ml per gram cellulose). After one more hour of continuous stirring and cooling in an ice bath, the cellulose xanthate is completely dissolved and the resulting product is ready, the so-called viscose solution. (Figure S2 d). The viscose solution is stored overnight at room temperature, to allow for ripening and therefore make it useable for the fiber spinning.


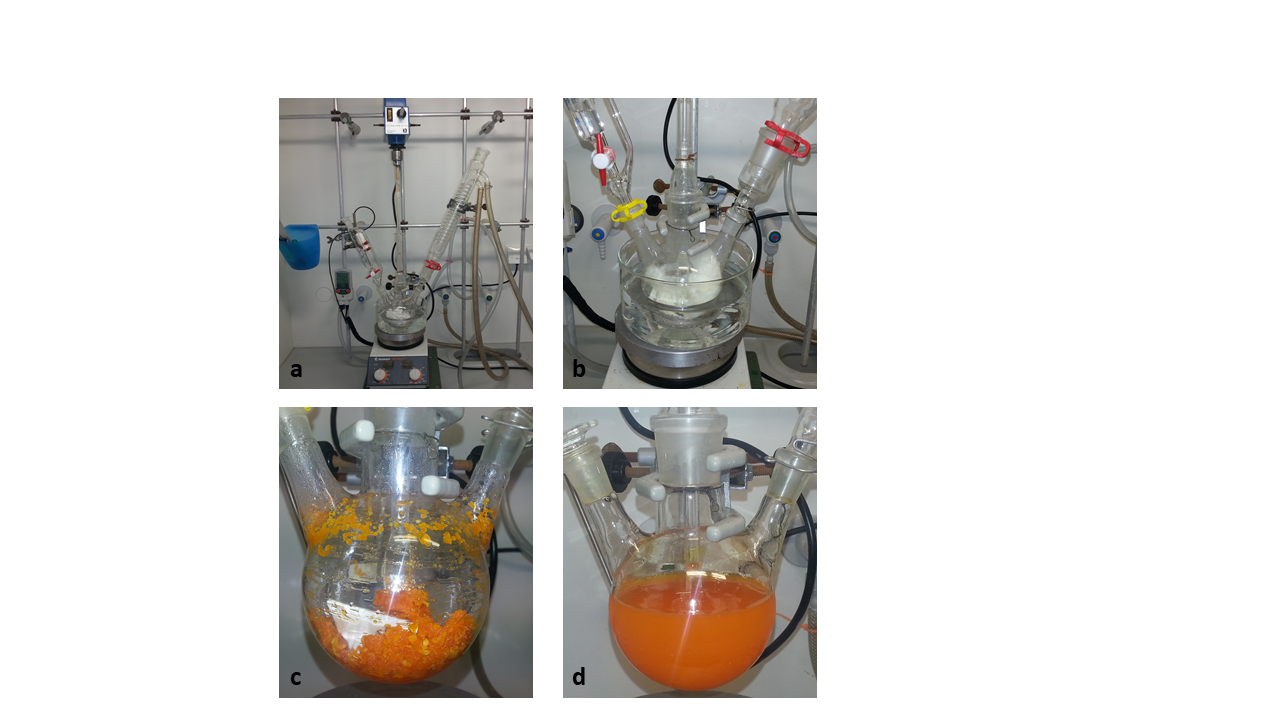


**Figure S2.** Overview on the xanthation of alkali cellulose. a: Preparation of the xanthation apparatus with the alkali cellulose deposited in the flask, b: Start of xanthation by adding CS_2_ dropwise, c: Cellulose xanthate after 3 hours of stirring, d: Final viscose solution

**1.2.3. Part 3: Fiber spinning**

**Aims**

Judging the influence of experimental parameters in viscose fiber spinning and connecting them to fiber quality and additive dosage.

**Theoretical background^8^**

Prior to fiber spinning, air bubbles and other solids in the cellulose xanthate solution must be removed since they would lead to a rupture of the fiber during spinning. Therefore, degassing and filtration of the xanthate solution is performed. Afterwards, the solution is pumped through a spinneret which is made of chemically inert alloys. The spinneret can consist of up to several thousands of nozzles with hole diameters between 40 to 200 microns. After passing the nozzle, the xanthate solution is injected into a temperature controlled regeneration bath. The regeneration bath contains sulfuric acid (usually 5-15 wt.%), and additives such as ZnSO_4_ and Na_2_SO_4_. Two processes, namely conversion to cellulose and coagulation, take place. Coagulation leads to a core-shell structure, which means that the outer parts of the injected xanthate solution solidifies, while the inner part is still in a liquid state. In addition, osmosis comes into play by to the removal of water due to presence of sodium sulfate. Subsequently, conversion to cellulose is induced by the sulfuric acid. The faster the regeneration, the thinner the shell and the worse the fiber quality is. Therefore, zinc sulfate is added in industry which forms a more stable xanthate, thereby slowing down regeneration speed (Scheme S6). As a consequence, a thicker shell is formed during coagulation, the fiber can be stretched to a larger extent, and the mechanical properties improve. The fibers are collected on rollers, and washed several times with hot water. Afterwards they are subjected to post treatment, which usually involves surface modification using fatty alcohols to facilitate further processing. After drying the final fibers are obtained.


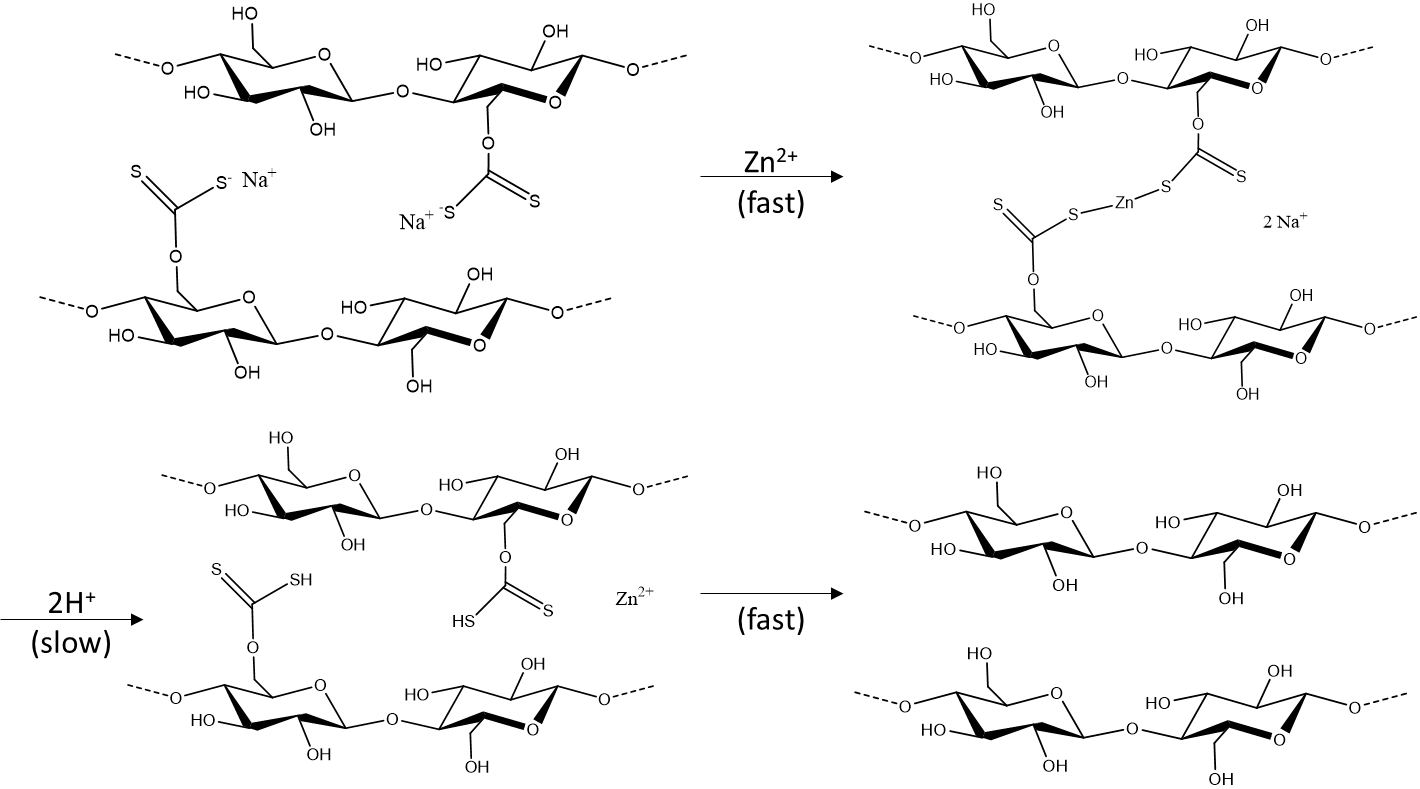


**Scheme S3.** Formation of zinc sulfate and subsequent regeneration to cellulose.

**Materials needed:**

Level 1:

Sulfuric acid 15 ml Harmful (strong acid)

Crystallizing dish 115mm 2

Magnetic stirrer 2

Syringe 1

Injection needles 2 / ∅ 45µm / 70 µm

DI water 135 ml

Protective gear (glasses, gloves, lab coat) for everybody

Level 2 (additional):

Syringe pump 1

Lab stirrer 1

Roller to collect the fiber 1

Sulfuric acid resistant plastic tube 1

Sulfuric acid 200 ml

DI water 1800 ml

**Before start:**

Make sure that everything you need is organized and available.

Inform yourself about the correct handling and the dangers of sulfuric acid solutions.

**To consider for the laboratory report:**

Determine the cellulose content in your viscose dope

Observe the ongoing reactions while the viscose solution is injected in the sulfuric acid solution

Describe the difference in the spun fibers before and after the washing step.

**Tasks:**

Determination of the cellulose content in the dope

The weight of a petri dish is determined before transferring a few ml (3-5) of viscose solution into the dish. The dish is slowly shaken, until the xanthate forms a thin film over the whole dish. The dish is placed in a drying oven at 60°C until the whole water is removed and a dry

solid xanthate film is obtained. This film is placed into sulfuric acid solution (10%) either with the dish or by peeling it off the dish.


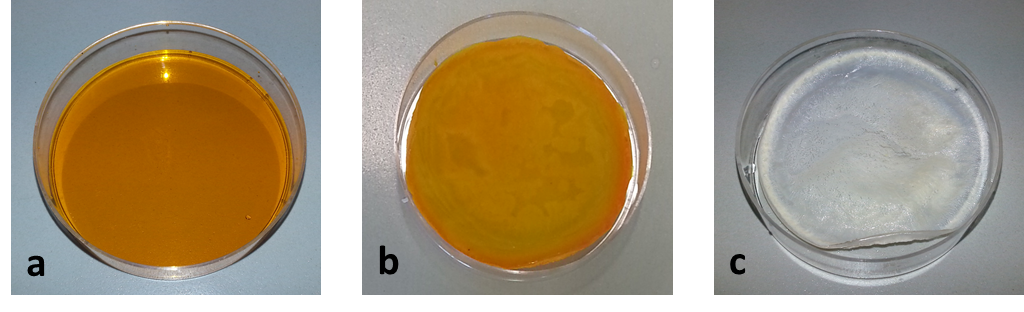


**Figure S3** Involved steps in the determination of the cellulose content in the spinning dope. a: viscose solution transferred into a petri dish, b: the cellulose xanthate film after drying, c: the remaining cellulose film after regeneration in H_2_SO_4_ and washing of the film

The regenerated cellulose film is extensively washed with water and dried again at 60°C until its dry mass remains constant. The cellulose content is determined as followed (16):

$Cellulose content \left[ \% \right]=\frac{Dry mass (cellulose film)}{Dry mass (cellulose xanthate film)}*100$ (16)

Fiber spinning (Level 1)

150 ml 10 wt% sulfuric acid solution are deposited in a petri dish (diameter 115 mm, glass) and the solution is heated to 50°C to accelerate the regeneration of the cellulose xanthate. The spinning dope is transferred into a syringe and the syringe is equipped with an injection needle (45 / 70 µm in diameter). The needle is placed in the sulfuric acid bath and the cellulose xanthate is injected. Fibers can be either collected after simple injection or by employing a slowly rotating stirring bar.

Regeneration is complete, when the color of the fiber changes from yellowish to white. Then, the fibers are immersed into a water bath for 15 minutes at elevated temperatures (T = 85°C). After washing has been completed, the fibers are dried at room temperature.

r
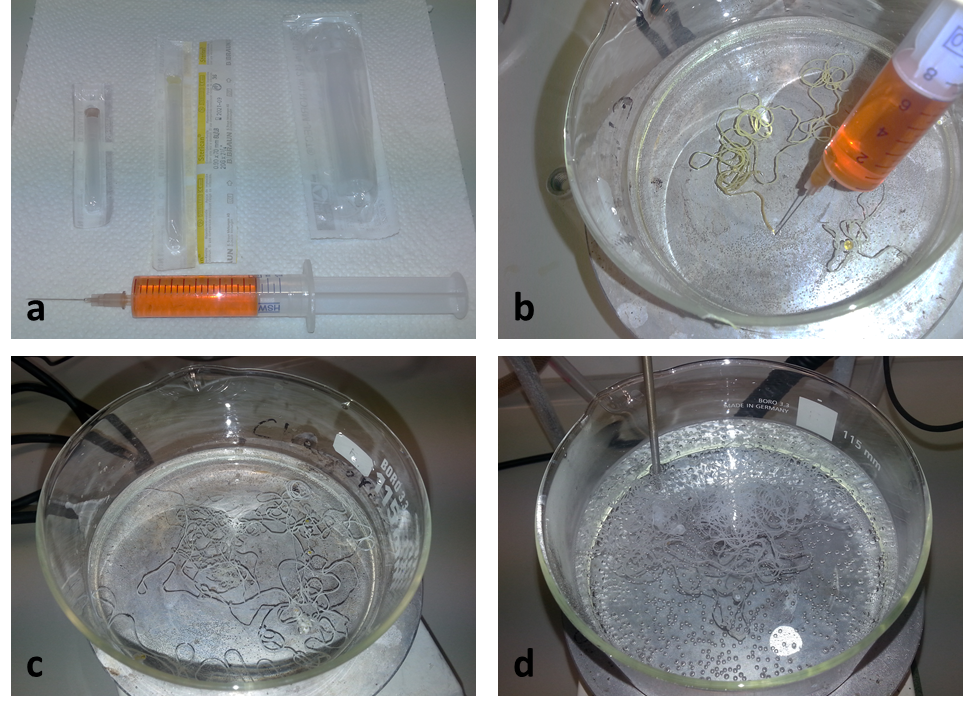


**Figure S4.** Different steps in the manual fiber spinning process. a: equipment, b: fiber spinning through manual injection, c: the fully regenerated fibers, d: fibers during washing in hot water.

Fiber spinning (Level 2)


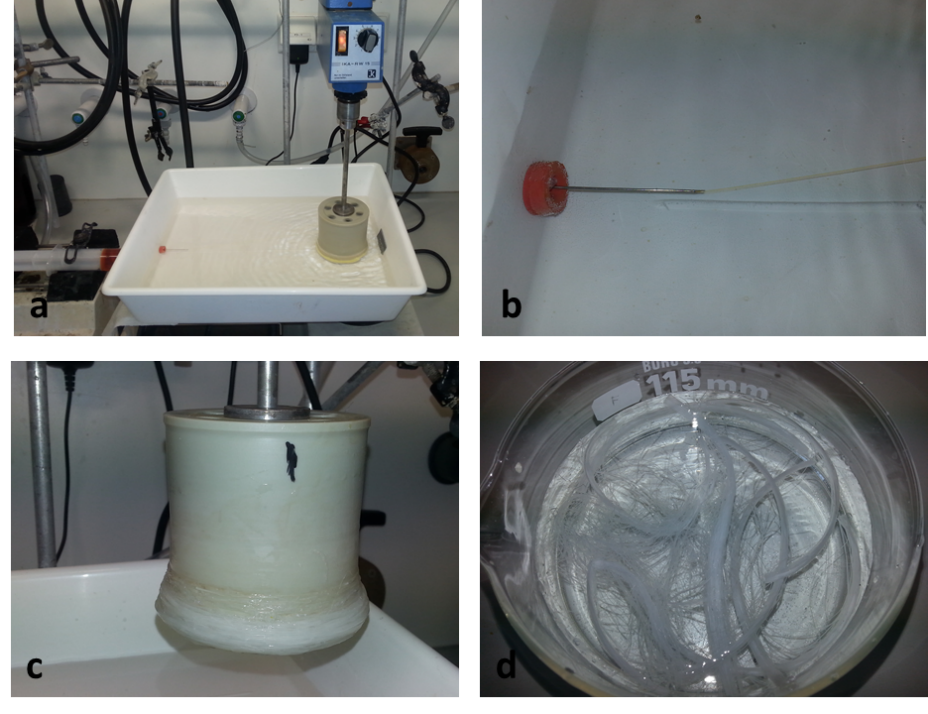
2 liters 10 wt% sulfuric acid solution are placed in a sulfuric acid resistant plastic tube (30 x 35 cm), equipped with a membrane. The fiber collector is placed into the sulfuric acid solution, opposite to the membrane. A syringe (20 ml) is filled with the viscose spinning dope and inserted in the syringe pump. An injection needle is fixed onto the syringe and placed into the sulfuric acid bath through the membrane. The viscose is injected continuously into the sulfuric acid using the syringe pump and the developing cellulose monofilament is collected on the slowly rotating fiber roller. It is important, to adjust the injection speed in order to avoid breaking of the fiber. After injecting the spinning dope, the fibers are stirred in the sulfuric acid until the regeneration of the cellulose is completed. The fibers can then be cut from the fiber collector and immersed in hot water (T=85°C) for 15 minutes. Afterwards, the water is exchanged, the fibers are immersed again for 15 minutes at 85°C and dried at room temperature.

**Figure S5**. Different steps in the manual fiber spinning process. a: equipment, b: fiber spinning through continuous injection with a syringe pump, c: fully regenerated fibers on the fiber collector, d: fibers during washing in hot water

**1.2.4. Part 4: Characterization**

**Aims of the task**

Correlate IR spectra of natural and man-made cellulose

Use IR spectroscopy to identify and assign intermediates in the viscose process

Evaluate fiber quality by optical microscopy and correlate it to the manufacturing process.

**Theoretical background^4, 9, 10^**

In nature, cellulose adopts a particular crystalline structure which is referred to as cellulose I. In this kinetically stabilized configuration, the reducing ends of cellulose are pointing at the same direction, which is called a parallel chain alignment. During the conversion to alkali cellulose the crystalline domains are partly destroyed and hydrogen bonds are broken up. After conversion to the xanthate and regeneration back to cellulose the thermodynamically stable form, so called cellulose II is obtained which is also partially crystalline. In this arrangement, the chains are aligned in an antiparallel manner i.e., the reducing ends of neighboring chains point at the opposite direction. As a consequence, the hydrogen bonding pattern is changing when going from cellulose I to alkali cellulose, cellulose xanthate and finally cellulose II. These differences in the arrangement of the chains can be tracked by simple analytical methods such as infrared spectroscopy. In IR spectroscopy, the vibrations of connected atoms can be detected via their absorption. A very prominent absorption band for cellulose is the OH stretching vibration, which appears in the range of 3200 to 3600 cm^-1^. In this area, differences in the hydrogen bonding system between cellulose I and II can be visualized and assigned to different modes. The broad band at 3600 – 3000 cm^-1^ in the alkali cellulose spectrum is shifted from 3500 cm^-1^ to 3650 cm^-1^ and the form changed to a continuous band. The higher intensity at 1640 cm^-1^ indicates the presence of additional OH stretching and deformation vibrations caused by bound water in the alkali cellulose. In addition, other functional groups in the course of the conversion can be tracked such as C-S and C=S vibrations in the xanthate and the various products described above. However, the IR analysis is not as straightforward as for the other sulfur containing products in the viscose process since there are many different compounds present in the solution.

The shape of fibers can influence their optical appearance such as shine but also their water uptake capability. Therefore, optical microscopy is an easy tool to observe differences in fiber appearance and to evaluate the influence of experimental variations. Parameters such as fiber diameter, transparency as well as homogeneity can be determined and compared on a qualitative way.

**Materials needed:**

Microscope slides

Tweezers

Optical microscope, equipped with a camera

IR spectrometer

**Before start:**

Inform yourself about the operating principle of the optical microscope and the IR spectrometer which are provided.

**Task:**

Investigate the form of the fibers spun with different needles and by different techniques (by hand, or semi-continuous with the syringe pump) and document the differences with the help of a microscope.

What is the difference in the optical appearance of the differently produced fibers and where do the difference originate from?

Calculate the diameter of the different fibers and explain why some are thicker/thinner than others.

Record IR spectra of the starting material (cotton), the stored alkali cellulose, a dried cellulose xanthate film and the regenerated fibers. Observe the difference in the spectra and try to assign the characteristic IR bands to the functional groups.

Explain the difference in the IR spectrum of the cellulose used as starting material and the cellulose obtained through the fiber spinning.

**1.3. Examples for exam questions:**

1. Name the main reactions in the Viscose process and complete the reaction scheme for the Viscose process.


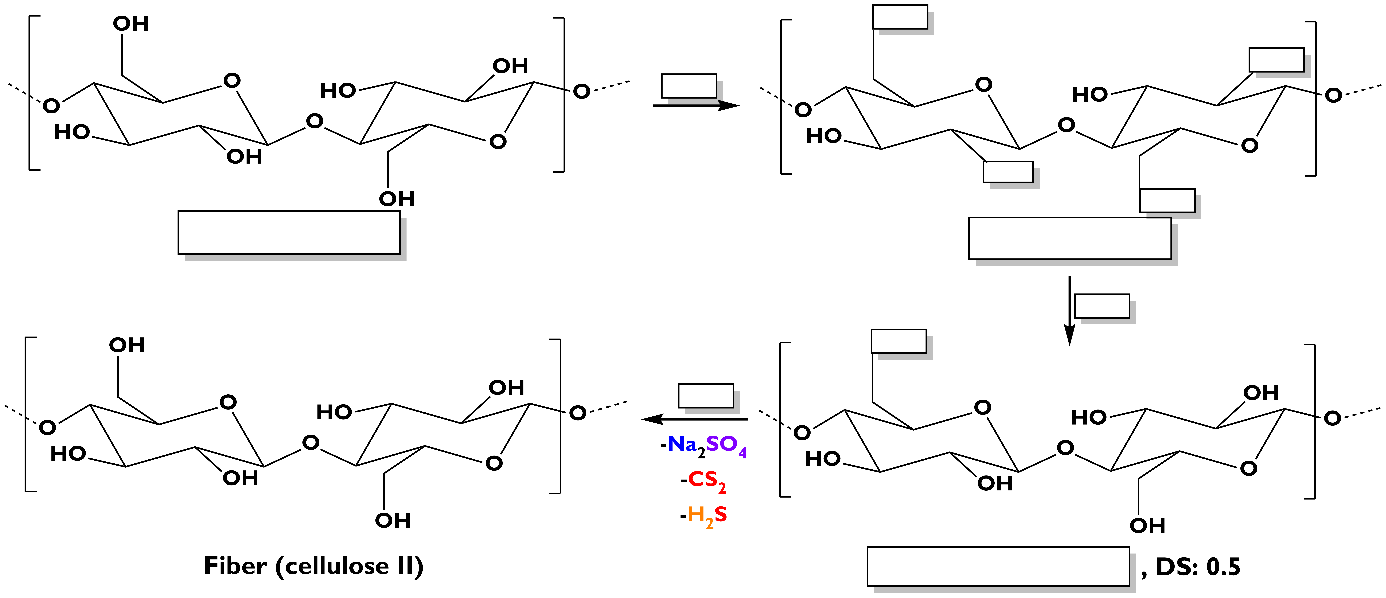


1. Explain shortly the preparation of alkali cellulose. Which reactions are going on during the synthesis and the ripening?
2. How does the alkali treatment affect the pulp fibers?
3. What is the gamma number?
4. What are primary and secondary reactions in the xanthation process, complete the reactions below and say if they are primary or secondary.

CS_2_ + ____-O^-^Na^+^ → Cell-________

CS_2_ + CS_2_O^2-^ → COS + _____

CS_2_ + ______ → HS-CSO^-^Na^+^

1. Which chemical processes happen during the ripening of cellulose xanthate and how do they relate to its spinnability?
2. Highlight the typical concentration of sulfuric acid in the spinning bath.

1-3% 5-15% 45-65%

1. What are the two processes taking place in the regeneration bath? Explain in 4 sentences what is meant and how they affect the fiber quality.
2. Describe in 2 sentences the role of Zn^2+^ cations in the fiber spinning.
3. What is the difference between cellulose I and II? How can differences be detected?

**1.4. Examples for exam questions – key:**

1. Name the main reactions in the Viscose process and complete the reaction scheme for the Viscose process.

Alkali cellulose synthesis, xanthation, regeneration, washing and post treatment of the fibers


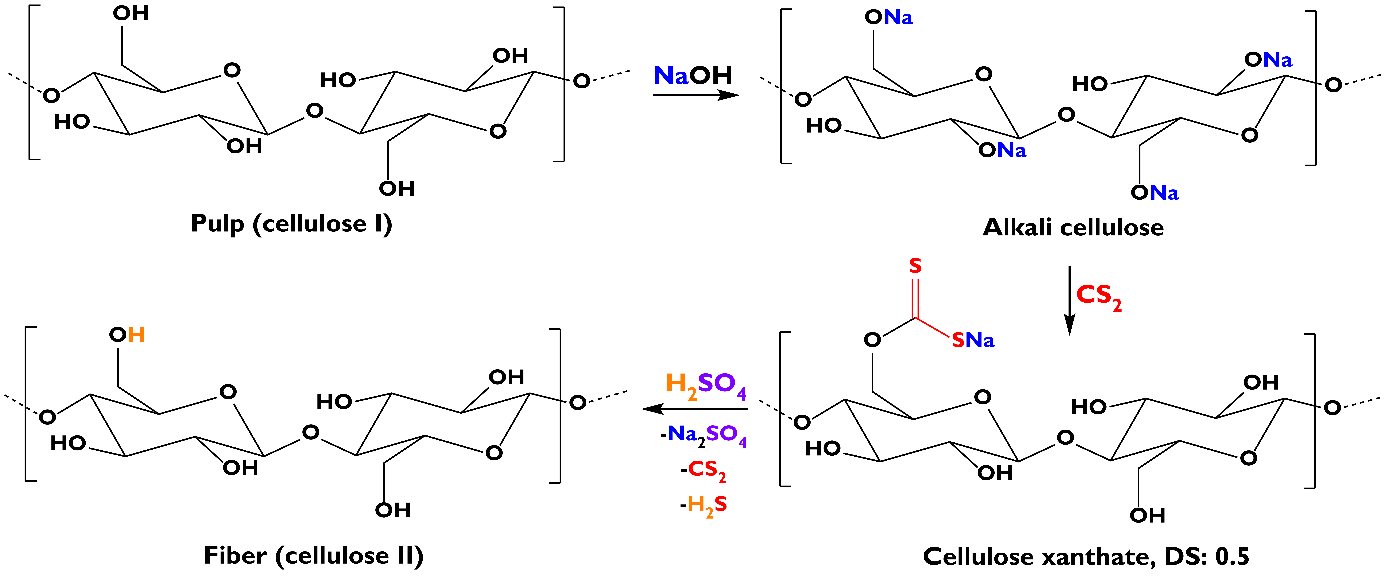


1. Explain shortly the preparation of alkali cellulose. Which reactions are going on during the synthesis and the ripening?

The main reactant that is used is sodium hydroxide, to formally form the sodium salt of cellulose, therefore pulp is mixed with 18-20 wt% NaOH to form a ca. 5 wt% suspension. During this reaction low molecular weight fractions of cellulose as well as the hemicelluloses are dissolved. During the ripening, a wealth of reactions take place, hydrogen bonds of the crystalline parts of the pulp are broken up and depolymerization of the cellulose macromolecules occurs.

1. How does the alkali treatment effect the pulp fibers?

The degree of polymerization is narrowed and the average DP decrease from the range 850-600 to 500-350

1. What is the gamma number?

The degree of xanthation is expressed in industrial terms as the so called gamma number. The gamma number is related to the degree of substitution via equation (E1):

$$\gamma=100*DS\left( X \right)$$

1. What are primary and secondary reactions in the xanthation process, complete the reactions below and say if they are primary or secondary.

In fact, a wealth of reactions proceed at this step, which can be distinguished in primary, secondary and side reactions. Primary reactions refer to those reactions where the desired cellulose xanthate is formed. The secondary reactions are those that occur with the formed products from the primary reactions

CS_2_ + Cell-O^-^Na^+^ → Cell-OCS_2_^-^Na^+^ primary

CS_2_ + CS_2_O^2-^ → COS + CS_3_^2-^ secondary

CS_2_ + Na^+^OH^-^ → HS-CSO^-^Na^+^ primary

1. Which chemical processes happen during the ripening of cellulose xanthate and how do they relate to its spinnability?

During ripening transxanthation reactions (either inter- or intramolecular) leads to preferred substitution at C6. In addition, also a partial cleavage of the xanthate groups occurs. Through this reactions, the xanthate substitution pattern along the cellulose chain gets homogenized and the chains are able to align with each other in a flow, what gives the fundamental condition for strong fibers.

1. Highlight the typical concentration of sulfuric acid in the spinning bath.

1-3% **5-15%**  45-65%

1. What are the two processes taking place in the regeneration bath? Explain in four sentences what is meant and how they affect the fiber quality.

Conversion to cellulose and coagulation, take place. Coagulation leads to a core-shell structure, which means that the outer parts of the injected xanthate solution solidifies, while the inner part is still liquid. Conversion to cellulose is induced by the sulfuric acid. The faster the regeneration, the thinner the shell and the worse the fiber quality is.

1. Describe in 2 sentences the role of Zn^2+^ cations in the fiber spinning.

Zinc sulfate is added to form a more stable xanthate, thereby slowing down regeneration speed. As a consequence a thicker shell can be formed during coagulation and the mechanical properties of the fibers increase.

1. What is the difference between cellulose I and II? How can differences be detected?

Cellulose in nature adopts a particular crystalline structure which is referred to as cellulose I, which is kinetically stabilized. In this configuration, the reducing ends of cellulose are pointing at the same direction, which is called a parallel chain alignment. In the arrangement of cellulose II (the thermodynamically stable form), the chains are aligned in an antiparallel manner, i.e. the reducing ends of neighboring chains point at the opposite direction. These differences in the arrangement of the chains can be tracked by simple analytical methods such as infrared spectroscopy.

ATR-IR spectra with the most important bands being assigned:


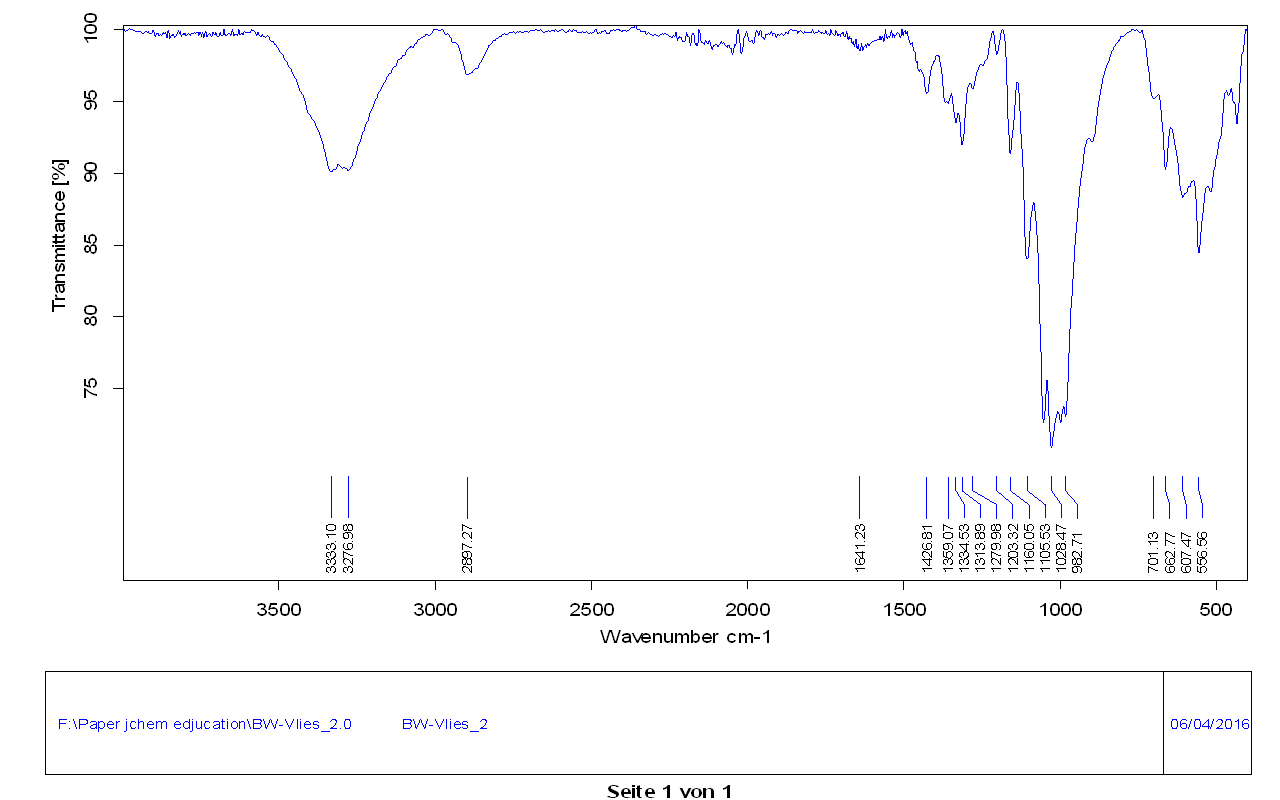


**Figure S6.** ATR-IR spectrum of the used cellulose source


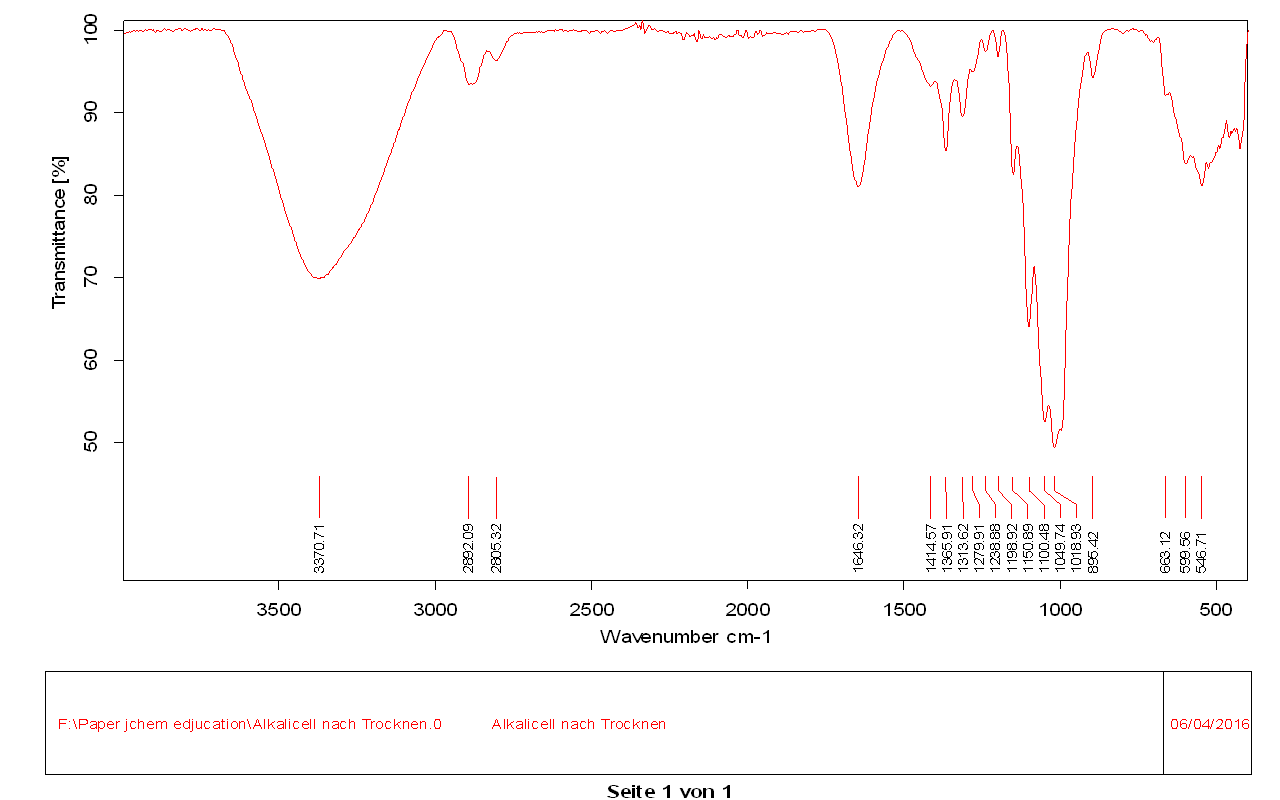


**Figure S7**. ATR-IR spectrum of the alkali cellulose

*
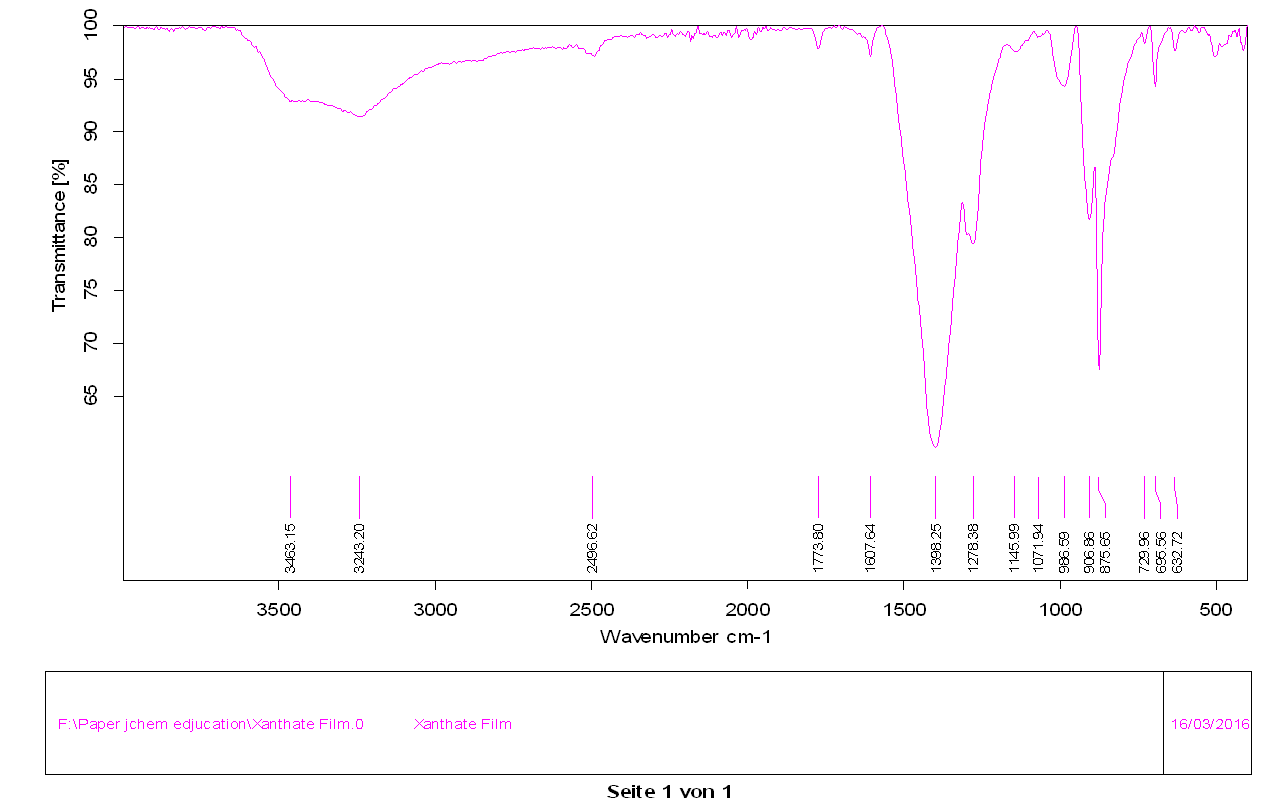
*

**Figure S8.** ATR-IR spectrum of the cellulose xanthate

*
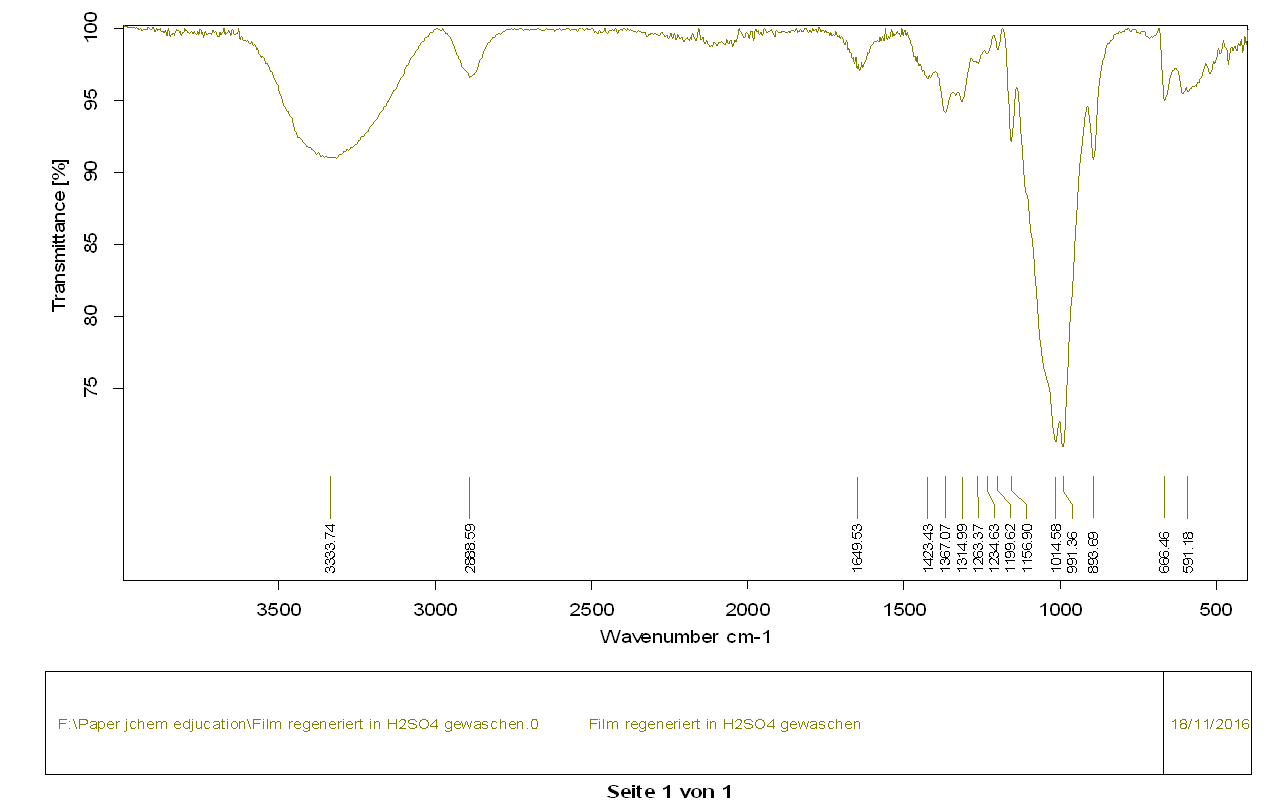
*

**Figure S9**. ATR-IR spectrum of the regenerated and washed cellulose fibers

**2. Virtual laboratory course on Viscose processing**

**2.1. Instructor notes**

For the virtual version, the teacher needs to be very flexible as the different students may have a different affinity to online tools and remote working. It requires to identify those students that have problems with the technology itself and to assist them with smooth solutions, providing guidance. This is particularly important for literature programs such as Mendeley or Citavi and how to use them most efficiently. For the writing parts, the teacher also needs to provide guidance as students will have problems to describe the main findings in their own language and often also with too much detail. For the laboratory design, the teacher needs to raise awareness for possible solutions that are doable in the respective environments. Large scale spinning devices are not standard equipment in most labs in the world. Awareness for safety is also an important issue for the students as well as how disposal of waste materials is considered in the design of the experiment. If available, the demonstration of the spinning experiment in real life for the students will have a positive impact on the learning effect.

For the virtual laboratory course basic information (Part 1-3) was provided on the individual steps (see below), which was the basis for the literature search to create a database. The database should contain between 25 and 50 entries from which the students have to identify the 3 most relevant papers, which they needs to summarize in 800-1000 words (Task 1). On the basis of this database, students need then to design (Task 2) the laboratory experiment and the required characterization (Task 3).

**2.2. Laboratory Handouts (virtual)**

**2.2.1. Part 1: Alkali cellulose synthesis**

**Theoretical background^1^**

The formation of alkali cellulose is the first step in the manufacturing of viscose fibers. The main reactant is sodium hydroxide to give the sodium salt of cellulose. In industry, standard dissolving pulp is mixed with NaOH to form a suspension. This suspension is exposed to a temperature between 25 and 55 °C for some hours. At this stage, the low molecular weight fraction of cellulose as well as the hemicelluloses dissolve and are removed by pressing the alkali cellulose. The removal of hemicellulose is essential for the resulting fiber quality since hemicellulose xanthate (which would be produced in the next step if they were not removed) are much more unstable than cellulose xanthate. This subsequently leads to formation of gels, thereby deteriorating the filterability of the obtained solutions. Further, hemicellulose incorporation into the cellulose fiber matrix during spinning leads to fiber with lower tenacity. After pressing, the alkali cellulose typically contains less than 3.5 wt.%. hemicelluloses, for speciality products even contents of below 1.6 wt.% are required. The pressing step also removes excess NaOH, which reacts with the CS_2_ in the xanthation step. After pressing, the alkali cellulose still contains bound and adsorbed NaOH. Then the alkali cellulose is subjected to pre-ripening, i.e. storage for a period of some hours under defined humidity control. During the pre-ripening a wealth of reactions take place, hydrogen bonds of the crystalline parts of the pulp are broken up and depolymerization of the cellulose macromolecules occurs. The most important reactions are depicted in Scheme S1.


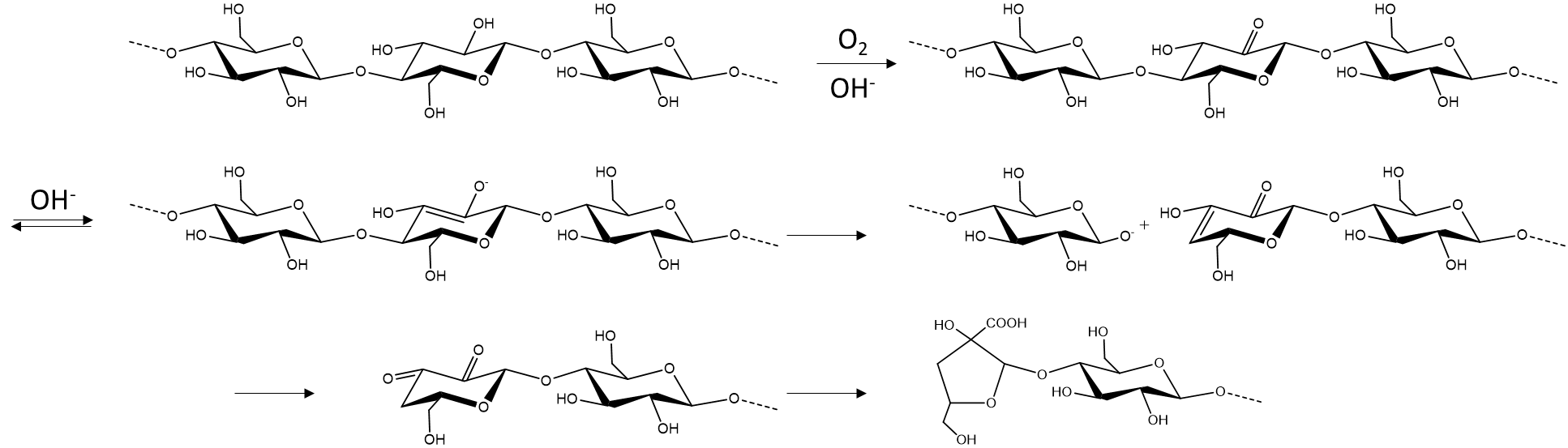


**Scheme S1**. Reactions during the processing of pulp to alkali cellulose


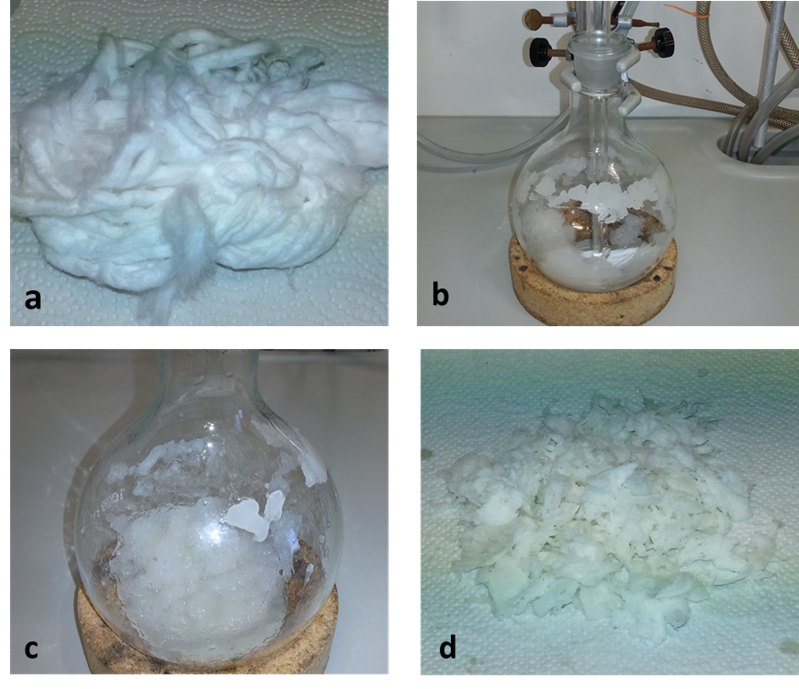


**Figure S1.** Illustration of alkali cellulose preparation. a: starting material-a cellulose source, b: stirring of the cotton fibers in NaOH solution, c: alkali cellulose before pressing, d: alkali cellulose after pressing.

**2.2.2. Part 2: Xanthation**

**Theoretical background^2-7^**

The next step in viscose synthesis is xanthation. In this step, the alkali cellulose is converted to the sodium cellulose xanthate. In industry, this exothermic reaction is done in huge cylinders where the CS_2_ is reacted in vaporous state. The degree of xanthation is expressed in industrial terms as the so called gamma number. The gamma number is related to the degree of substitution (DS) via equation (1):

$\gamma=100*DS\left( X \right)$ (1)

In industry, cellulose xanthate with a gamma value of around 50 is usually manufactured for fiber spinning, which means that in average just 1 out of two AGU is substituted with xanthates. Higher gamma numbers can be realized if subsequent additions of CS_2_ are performed, in lab scale even full substitution (gamma 300) can be realized.

In fact, a wealth of reactions proceeds at this step, which can be classified into primary, secondary and side reactions. Therefore 30 up to 50 wt.% of CS_2_ related to cellulose are needed in industrial scale to accomplish the reaction to the desired product. Primary reactions refer to those reactions where the desired cellulose xanthate is formed (eq 2-5).

CS_2_ + Cell-O^-^Na^+^ → Cell-OCS_2_^-^Na^+^  (2)

CS_2_ + Na^+^OH^-^ → HS-CSO^-^Na^+^ (3)

HCS_2_O^-^Na^+^ + Cell-OH → Cell-OCS_2_^-^Na^+^ + H_2_O (4)

HCS_2_O^-^Na^+^ + Na^+^OH^-^ → CS_2_O^2-^ + 2 Na^+^ (5)

The secondary reactions are those that occur with the formed products from the primary reactions such as the thiol and the dithiocarbonate. The latter for instance reacts with CS_2_ to form COS, which in turn reacts with NaOH to the carbonate under release of thiols, which then are further converted to the trithiocarbonate (eq 6-10).

CS_2_ + CS_2_O^2-^ → COS + CS_3_^2-^ (6)

COS + 3 OH^-^ → CO_3_^2-^ + SH^-^ + H_2_O (7)

CS_2_ + SH^-^ → CS_3_H^-^ (8)

CS_3_H^-^ + OH^-^ → CS_3_^2-^ + H_2_O (9)

CS_2_O^2-^ + 2 OH^-^ → 2 SH^-^ + CO_3_^2^ (10)^-^

In addition, the reaction of NaOH and CS_2_ (side reactions) leads to a variety of different sulfur containing compounds such as sodium carbonate, sodium thiolate, sodium trithiocarbonate, hydrogen sulfide, and sodium sulfide. (eq 11-14)

CS_2_ + 4 NaOH → Na_2_CO_3_ + 2 NaHS + H_2_O (11)

2 CS_2_ + 4 NaOH → Na_2_CO_3_ + Na_2_CS_3_ + H_2_S + H_2_O (12)

3 CS_2_ + 6 NaOH → 2 Na_2_CS_3_ + Na_2_CO_3_ + 3 H_2_O (13)

5 CS_2_ + 12 NaOH → Na_2_S + 2 Na2CO3 + 3 Na_2_CS_3_ + 6 H_2_O (14)

From these reactions, it is also obvious why the pressing step after alkali cellulose preparation is so important. Every mol of NaOH can consume CS_2_ which is an economically important factor. After the xanthation has been finished, the cellulose xanthate is diluted with NaOH, a usual ratio is 1:4 (1 part alkali cellulose, 4 parts NaOH) to form the so called viscose. Then the viscose (before xanthate) is transferred to a tank, where the ripening takes place. The conditions for the ripening are crucial: cellulose xanthate decomposes at higher temperatures but also at the lower temperatures transxanthation reactions occur, which are, however, beneficial for fiber spinning. In order to obtain mechanically strong fibers, the macromolecules must be of similar size and in addition, they must be able to align with each other under flow conditions. This can be realized much easier when the xanthate substitution pattern along the cellulose chain is homogeneous. Here, it is important to know that the thermodynamically most stable position for the xanthate is the C6 position; therefore any rearrangements, i.e. transxanthation reactions (either inter- or intramolecular) lead to preferred substitution at C6. In addition, also a partial cleavage of the xanthate groups occurs whereas hydrolysis is ca. times faster at position C2 and C3 compared to C6, thereby also contributing to a final cellulose xanthate spinning dope where the xanthate groups are preferentially located at C6. An overview is presented in Figure S2 and S3.


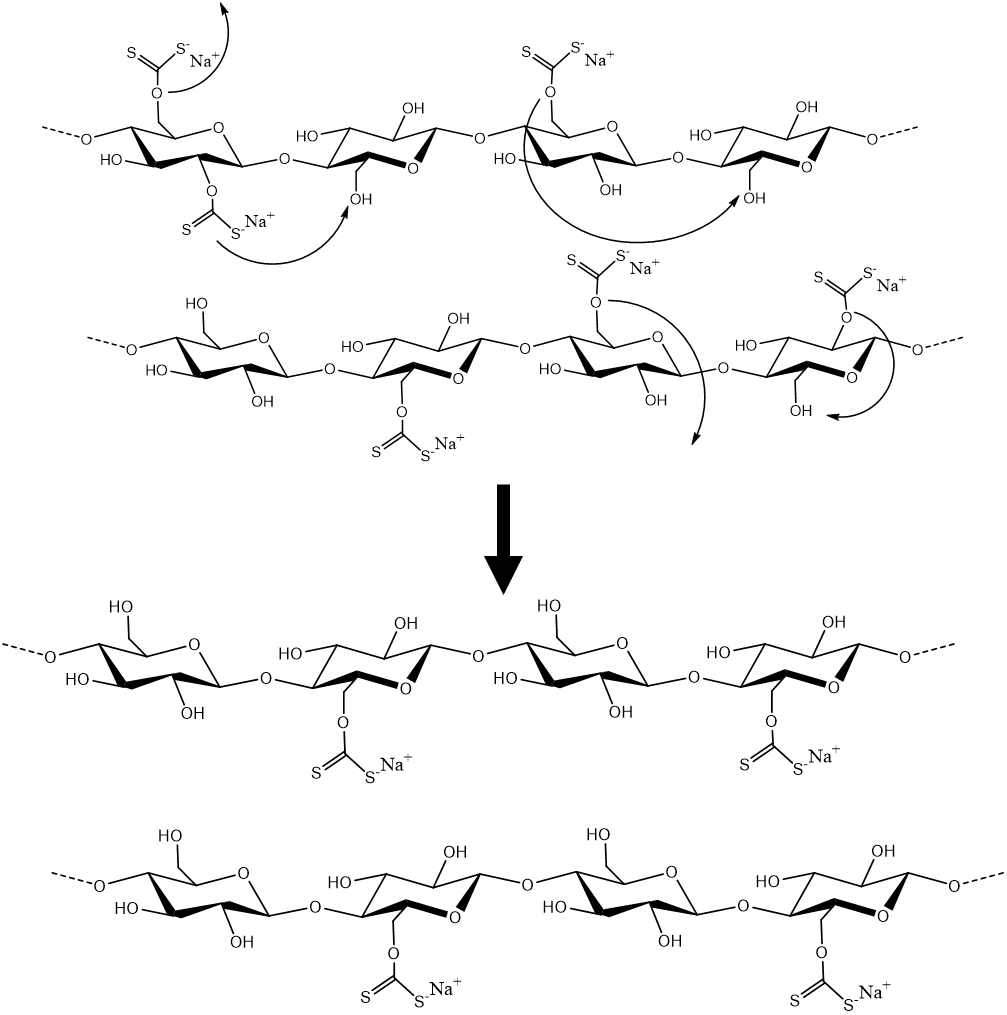


**Figure S2**. Overview of cleavage and transxanthation reactions


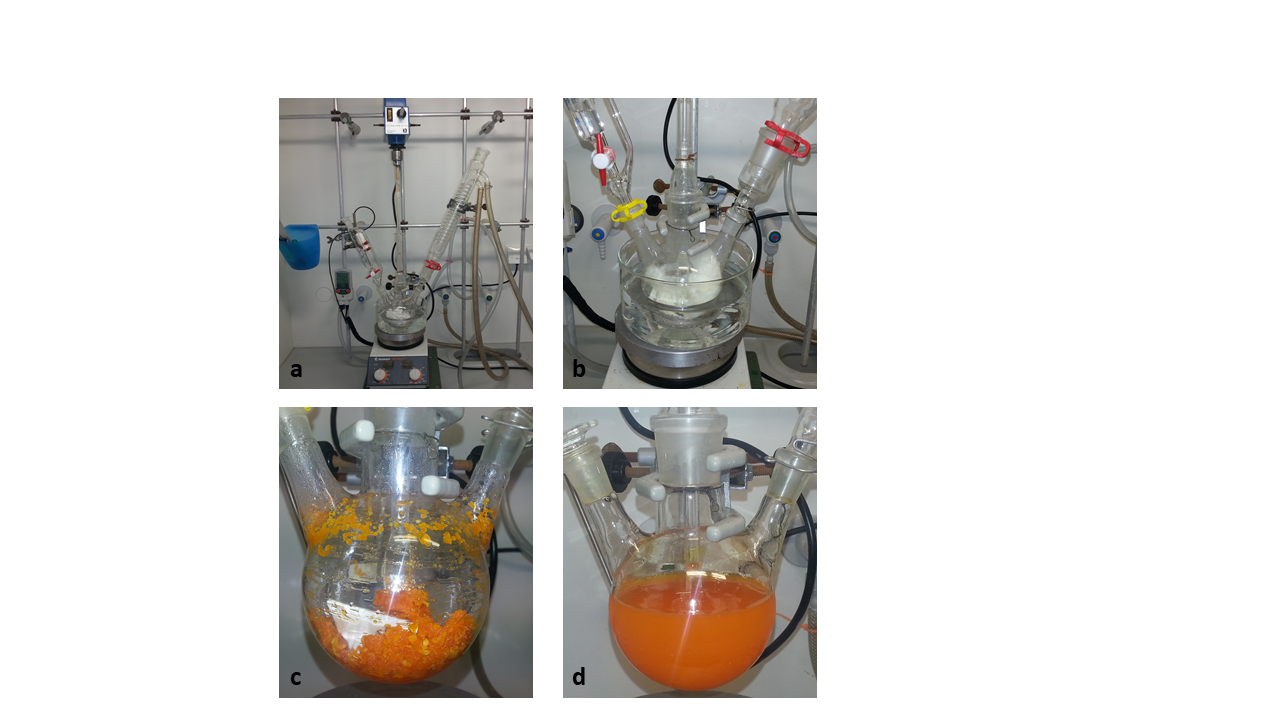


**Figure S3.** Overview on the xanthation of alkali cellulose. a: Preparation of the xanthation apparatus with the alkali cellulose deposited in the flask, b: Start of xanthation by adding CS_2_ dropwise, c: Cellulose xanthate after several hours of stirring, d: Final viscose solution

**2.2.3. Part 3: Fiber spinning**

**Theoretical background^8^**

Prior to fiber spinning, air bubbles and other solids in the cellulose xanthate solution must be removed since they would lead to a rupture of the fiber during spinning. Therefore, degassing and filtration of the xanthate solution is performed. Afterwards, the solution is pumped through a spinneret which is made of chemically inert alloys. The spinneret can consist of up to several thousands of nozzles with hole diameters in the micron range. After passing the nozzle, the xanthate solution is injected into a temperature-controlled regeneration bath. The regeneration bath contains sulfuric acid, and additives such as ZnSO_4_ and Na_2_SO_4_. Two processes, namely conversion to cellulose and coagulation, take place. Coagulation leads to a core-shell structure, which means that the outer parts of the injected xanthate solution solidifies, while the inner part is still in a liquid state. In addition, osmosis comes into play by to the removal of water due to presence of sodium sulfate. Subsequently, conversion to cellulose is induced by the sulfuric acid. The faster the regeneration, the thinner the shell and the worse the fiber quality is. Therefore, zinc sulfate is added in industry which forms a more stable xanthate, thereby slowing down regeneration speed (Figure S4). As a consequence, a thicker shell is formed during coagulation, the fiber can be stretched to a larger extent, and the mechanical properties improve. The fibers are collected on rollers, and washed several times with hot water. Afterwards they are subjected to post treatment, which usually involves surface modification using fatty alcohols to facilitate further processing. After drying the final fibers are obtained.


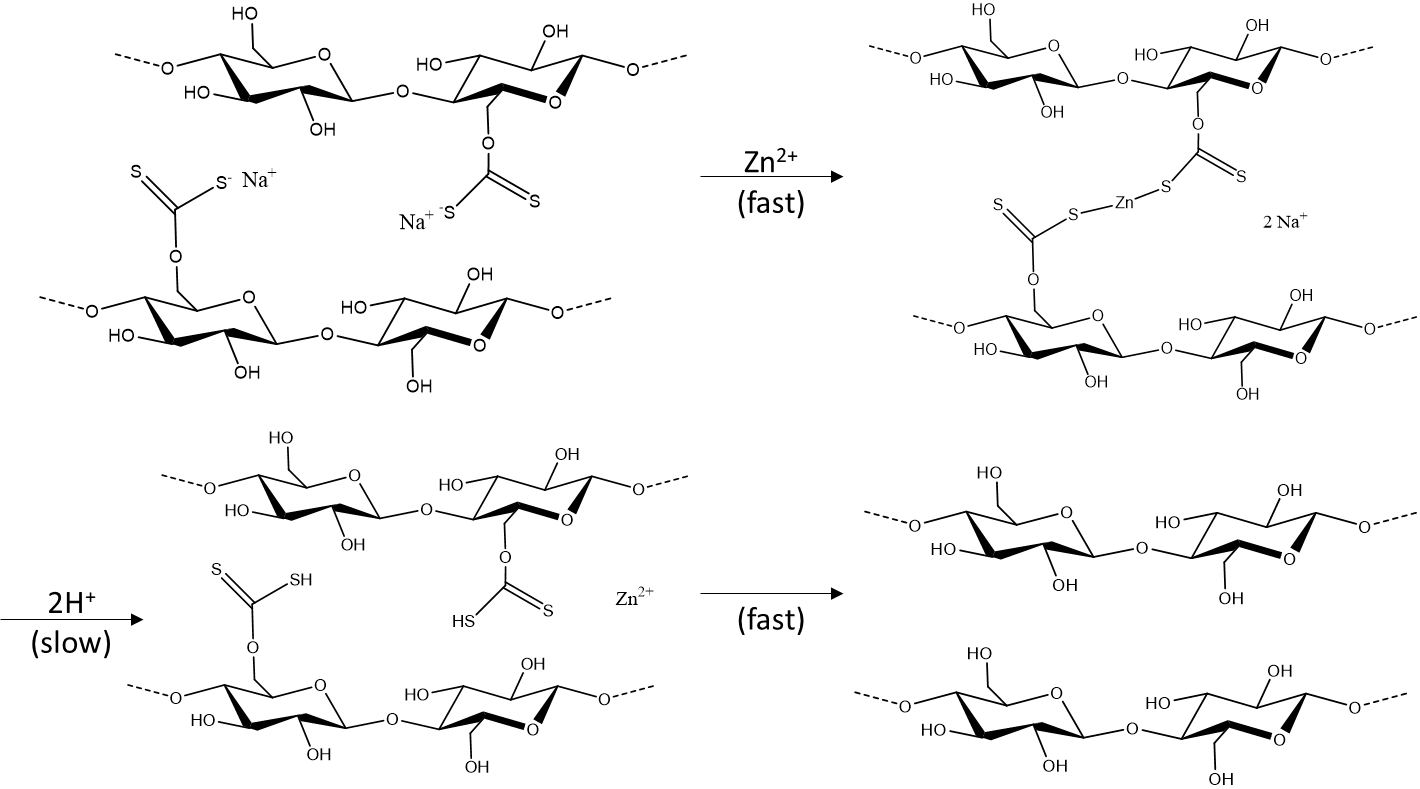


**Figure S4.** Formation of zinc sulfate and subsequent regeneration to cellulose.

**2.3. Tasks for the virtual laboratory course**

**Task 1: Literature research on viscose processing**

-Identify the crucial literature using either SciFinder, Scopus or Web of Knowledge and create a database using a literature management software (e.g. Mendeley or Citavi). Do not use any webpages to derive information as these are not subject to peer review and cannot be considered trusted sources. You may search of course videos/webpages to get an idea of the process steps.

-Focus in the literature research on practical aspects, e.g. experimental and processing conditions during all steps of viscose manufacturing.

-Write a short summary (800-1000 words) of the 3 most relevant papers and their connection to the lab exercise

**Task 2: Designing the lab experiment.**

-Develop a procedure how to perform the experiments and give a detailed description in the same manner as in a scientific paper. Use the information from your literature search and take the pictures given in this instruction as inspiration. Try to provide schemes and illustrations.

Consider following points in particular:

-What infrastructure do you need for performing the experiments (space, equipment)?

-Which chemicals/materials do you need? How dangerous are these?

-Which protection gear do you need?

-How do you dispose waste?

**Task 3: Characterization of your materials**

-Provide a strategy how to analyze your materials at all stages of the experiments. Use the literature you found as the basis to develop the procedure.

**References**

(1) Sixta, H. *Handbook of Pulp*. Wiley: New York, 2008.

(2) Andrews, D. A.; Hurtubise, F. G.; Krassig, H. The Presence of Monothiocarbonate Substituents in Cellulose Xanthates. *Can. J. Chem.* **1960,** *38*, 1381-1394.

(3) Hovenkamp, S. G. Sodium dithiocarbonate as a by-product in xanthating reactions. A contribution to the chemistry of viscose. *J. Polym. Sci.* **1963,** *Pt C. No. 2*, 341-355.

(4) Ogura, K.; Sobue, H. Studies on the derivatives of sodium cellulose xanthate. Part I. Infrared absorption spectra and characteristic frequencies of C-S and C=S groups in sodium cellulose xanthate and its stable derivatives. *J. Polym. Sci, Part B: Polym. Lett.* **1968,** *6*, 63-67.

(5) Dautzenberg, H.; Philipp, B. Über Bildungsweise und Verhalten des Natriumdithiocarbonats. *Z. Anorg. Allg. Chem.* **1970,** *375*, 113-123.

(6) Wöss, K.; Weber, H.; Grundnig, P.; Röder, T.; Weber, H. K. Rapid determination of γ-value and xanthate group distribution on viscose by liquid-state 1H NMR spectroscopy. *Carbohydr. Polym.* **2016,** *141*, 184-189.

(7) He, L.; Hu, H.-C.; Chai, X.-S. A Real-Time Technique for Monitoring Cellulose Dissolution during the Xanthation Process. *Industrial & Engineering Chemistry Research* **2016,** *55*, 10823-10828.

(8) Woodings, C. *Regenerated Cellulose Fibres*. Woodhead Publishing: 2001.

(9) Klemm, D.; Heublein, B.; Fink, H.-P.; Bohn, A. Cellulose: Fascinating Biopolymer and Sustainable Raw Material. *Angew. Chem. Int. Ed.* **2005,** *44*, 3358–3393.

(10) Weißl, M.; Niegelhell, K.; Reishofer, D.; Zankel, A.; Innerlohinger, J.; Spirk, S. Homogeneous cellulose thin films by regeneration of cellulose xanthate: properties and characterization. *Cellulose* **2018,** *25*, 711-721.
